# Concepts and Synonymy in the UMLS Metathesaurus 

**Published:** 2009-09-14

**Authors:** Gary H. Merrill

**Affiliations:** 1 GlaxoSmithKline, Research Triangle Park, NC, USA, 27709

## Abstract

This paper advances a detailed exploration of the complex relationships among terms, concepts, and syn­onymy in the UMLS (Uniﬁed Medical Language System) Metathesaurus, and proposes the study and under­standing of the Metathesaurus from a model-theoretic perspective. Initial sections provide the background and motivation for such an approach, and a careful informal treatment of these notions is offered as a con­text and basis for the formal analysis. What emerges from this is a set of puzzles and confusions in the Metathesaurus and its literature pertaining to synonymy and its relation to terms and concepts. A model theory for a segment of the Metathesaurus is then constructed, and its adequacy relative to the informal treatment is demonstrated. Finally, it is shown how this approach clariﬁes and addresses the puzzles educed from the informal discussion, and how the model-theoretic perspective may be employed to eval­uate some fundamental criticisms of the Metathesaurus. For users of the UMLS, two signiﬁcant results of this analysis are a rigorous clariﬁcation of the different senses of synonymy that appear in treatments of the Metathesaurus and an illustration of the dangers in computing inferences involving ambiguous terms.

## Introduction

The UMLS (Uniﬁed Medical Language System) Metathesaurus is a rich and powerful resource in biomedical informatics, ﬁnding application in such areas as clinical coding, enhanced information retrieval, knowledge exploration, and data mining and inferencing. Fundamental to these roles is its representation of synonymy and concepts that transcends multiple “source vocabularies” and seeks to provide a coherent and uniﬁed view of the biomedical domain.  1 

The approach taken to concepts and synonymy in the UMLS is described in online UMLS Metathesaurus documentation (NLM (2008)) and in a series of short papers over a span of approximately ﬁfteen years. The Metathesaurus documentation itself, while containing some degree of technical detail and a few examples, leaves many questions unanswered. Moreover, this literature is sometimes difﬁcult to grasp for those lacking experience or familiarity with relational data bases and data models; and at times it requires some degree of sophistication in such areas as controlled vocabularies, thesauri, and computational linguistics. The published papers function primarily as brief reports in which techniques or studies are summarized or sketched, and those devoted to a conceptual presentation lack detail and rigor.  2 They tend to provide a view either at the broad and informal conceptual level, at one extreme, or at the implementation level in terms of data models or object models at the other. It is difficult to gain a comfortable understanding of the critical subjects from the “conceptual” documentation, while delving into the detailed technical treatments is both demanding and time-consuming – and at times confusing, since some of these are offered as alternatives to (or improvements to, or evolutions of) others; and there is some degree of inconsistency among the published papers and the UMLS documentation. 

If we ask the question “What are the fundamental principles that govern the use of – and relations among – concepts, terms, and synonyms in the Metathesaurus?", then on the basis of current Metathesaurus documentation and publications it is virtually impossible to answer this question in a manner that is both accurate and fairly concise — or in one which is precise and accurate while not being closely bound to one or another data model or application implementation. At the heart of this situation is the fact that currently there does not exist a formally well-deﬁned and implementation-independent framework (such as the description logic underlying SNOMED-CT 3) in which principles concerning the fundamental elements of the Metathesaurus model can be stated, or in which questions about it can be phrased. 

As an example, consider that the treatment of synonymy in UMLS publications by NLM (U.S. National Library of Medicine) authors is particularly perplexing. It is repeatedly asserted that synonymy in the Metathesaurus is a relation among terms. For example, 

UMLS 1

“Identical character strings in the same language, disregarding case, are grouped into string classes; lexical variants are grouped into term classes; and synonymous names are joined in a concept.

Synonyms are lexically dissimilar names which carry the same meaning; sets of synonyms form equivalence relationships to each other.

The complete set of preferred names, lexical variants, and synonyms form a concept or conceptual group.”

Schuyler, et al. (1993)

UMLS 2

“X is a synonym of Y, if any simple sentence S containing X entails and is entailed by another sentence Sf, which is identical to S except that X is replaced by Y.

As noted earlier, an entry in the Metathesaurus may consist of a number of terms which form a synonym class. By the deﬁnition of synonymy given above, each of the terms represents exactly the same meaning.”

McCray and Nelson (1995)

UMLS 3

“The UMLS Metathesaurus is organized by concept or meaning, which is a cluster of synonymous terms. Concepts are identiﬁed by a concept unique identiﬁer (CUI) which is needed to instantiate Concept objects. ”

Bodenreider (2001)

UMLS 4

“The single CUI represents the Metathesaurus assertion that all these terms are synonymous.”

Hole, et al. (2004)

UMLS 5

“Synonymy is a central notion in the organization of a concept-based terminology. Names that have the same meaning (i.e., synonyms) are encompassed by the same concept. In linguistics, synonymy can be deﬁned as follows: X and Y are synonyms if any sentence S1 containing X is equivalent to another sentence S2, which is identical to S1 except that X is replaced by Y. More simply put, X and Y are synonyms if they can be used interchangeably in all circumstances. For instance, ‘celiac disease’ and ‘gluten enteropathy’ are synonyms as the two sentences (and all other sentences differing only by the two concept names): ‘Celiac disease is a chronic familial disorder associated with sensitivity to dietary gluten’ and ‘Gluten enteropathy is a chronic familial disorder associated with sensitivity to dietary gluten’ are equivalent in meaning.”

Fung, et al. (2005)

UMLS 6

“In effect, the UMLS Metathesaurus is a terminology integration system, in which synonymous terms from various terminologies are clustered into concepts, allowing for seamless mapping between terms from different terminologies through a UMLS concept.”

Bodenreider (2008)

Thus we have a rather uniform view across the years, during which the Metathesaurus has been developed and expanded, that the Metathesaurus consists of terms (occurring in “sources” or “vocabularies”) which are related to concepts (construed as the “meanings” of terms) and which are in some circumstances related to one another by synonymy. 

In fact, there is something of a shift over the years from a strongly term-centric view of the Metathesaurus (as exempliﬁed by Schuyler, et al. (1993) and McCray and Nelson (1995)) to a more concept-oriented view (as found in Bodenreider (2001, 2008)). And although the UMLS and Metathesaurus are not mentioned explicitly in McCray (2006), several of its source vocabularies are, and it takes a strongly concept-oriented view of organizing and categorizing scientiﬁc knowledge. However, it is difﬁcult to determine whether this apparent shift to thinking of the Metathesaurus as a meta-ontology rather than a meta-thesaurus is simply a change in emphasis or perspective, or rather is a more fundamental change in the semantics (or intended semantics) of the Metathesaurus. Nontheless, the Metathesaurus (particularly in its documentation and in the minds of most of its users) retains a fundamental focus on “terms” as these appear in “vocabularies”, and concepts are seen as appearing in a distinct “layer” above this more fundamental one. As a consequence, even the picture sketched in the citations above leads to some puzzling questions. 

### 1.1 Two illustrative puzzles 

The ﬁrst puzzling question pertains to whether there is in fact a single sense of synonymy at play in the Metathesaurus, or rather two or more senses of synonymy – and the related question as to precisely what kinds of things enter the synonymy relation (or relations).^4^ In McCray and Nelson (1995), early sections explicitly describe the synonymy relation in a classic manner as being supported by interchange principles — and hence as an equivalence relation (reﬂexive, symmetric, and transitive). But in a later section, it is said that 

UMLS 7

“Either a source vocabulary lists two terms as synonyms, or lexical matching techniques suggest terms for inclusion in a synonym class .....  In either case, subsequent review by a Metathesaurus editor determines whether the terms are actually synonyms or not.”

McCray and Nelson (1995)

and this is followed by an example in which the terms ‘hepatoma’ and ‘hepatocellular carcinoma’ are as­serted as synonymous in SNOMED-CT, but are not regarded as synonymous in the Metathesaurus (i.e., they are “treated as separate concepts” in the Metathesaurus).^5^  Such a treatment suggests that there is a difference between apparent synonymy and actual synonymy, or perhaps between a kind of “local synonymy” (e.g., within a source such as SNOMED-CT) and source-transcendant “actual synonymy”. Thus although a source may list two terms as synonyms, it may be the case that they are not really synonyms – leading us to wonder what ‘t_1_ is really a synonym of t_2_’ means. Perhaps this could be explained by taking the position that it is occurrences of terms (within a source) that enter the synonymy relation in a funda­mental sense while terms themselves enter it in a source-transcendant sense – or fail to enter it even if their occurrences have done so in one or more sources. But the details of such relationships are unclear at that point, and the implication that the “true” sense of synonymy must be determined by manual intervention and “review” is somewhat concerning in what otherwise appears as a formal account of the relation. UMLS 1 and UMLS 5 seem to state a clear formal criterion of synonymy, but UMLS 7 appears to take it back. 

The second puzzle involves the notion of ambiguous term and whether it is even possible for there to be ambiguous terms given how the relation between concepts and synonymy is characterized in Metathe­saurus literature. UMLS 3 is followed immediately by the account that 

UMLS 8

“A given term may have several meanings and belong to several concepts, which prevents a term from unambiguously instantiating a concept.”

Bodenreider (2001)

and a similar view of concepts and terms is taken more recently in Nelson, et al. (2006) and throughout the NLM literature on the UMLS:

UMLS 9

“Each concept record contains the strings of alphanumeric characters and terms that express the meaning of the concept. Strings that are lexical variants of each other (that is, identical after a series of well-deﬁned manipulations that can be done computationally, e.g., making all characters lower case, putting all words in a deﬁned order, and changing all plural forms to singular) are grouped together as a single term. One string is designated, by convention, as the preferred form of that term.”

Nelson, et al. (2006)

Here it is clear that a “single term” is not simply a string, but is in fact an equivalence class of strings – each of which is regarded as one “form” of the term. For example the terms ‘Thrombocytopenia’, ‘Thrombocytopaenia’, ‘THROMBOCYTOPENIA NOS’, and ‘Thrombocytopenia, NOS’ are all regarded as the “same term” in the Metathesaurus, being members of the lexical equivalence class whose identiﬁer is L0040034. 

Now UMLS 8 acknowledges the existence of ambiguous terms and immediately gives rise to a puzzle: If a concept forms an equivalence class of terms under the synonymy relation, is a concept closed under that relation? That is, if t_1_ is in concept C and t_2_ is a synonym of t_1_, is it therefore the case that t_2_ is in C? In the common understanding of synonymy expressed in UMLS 1 – UMLS 6, this would be the case. 

Another way of putting this is to say that in the common understanding of the synonymy relation it is a genuine equivalence relation, and hence transitive. Moreover, given the deﬁnition of synonymy in terms of interchange (as in UMLS 2), the transitivity of synonymy is *provable*. Unfortunately, it would appear to follow from this that UMLS 8 is false since if (a) a concept is a set of synonymous terms and (b) all of the synonyms of a term are in the same concept(s) as that term itself, then (c) there cannot be two distinct concepts that contain the same term: every concept that contains the term t will also contain all the synonyms of t, and so any term t can be in only one concept. Ambiguity is impossible if concepts are closed under synonymy. 

Consequently, if UMLS 8 is indeed true, then either ‘synonym’, ‘is synonymous’ and ‘synonymy’ must mean something different from what UMLS 1 -UMLS 6 seem to be explicitly asserting, or ‘term’ must mean something different than we are taking it to mean (this is hinted at in UMLS 8 where a term itself begins to appear as some kind of equivalence class, and it is even more explicit in UMLS 9), or perhaps synonymy is not a relation among terms (as we think we understand that notion). Some other questions that immediately arise from this situation are “What does it mean for ambiguous terms to be synonymous?” and “Is it even possible for ambiguous terms to be synonymous?” and “If it is not, then how can ambiguous terms ever appear in concepts since concepts are sets of synonyms?”. 

To be sure, there may be several ways out of these puzzles. For example, again, perhaps it is not terms (in our somewhat naive understanding of this word) that enter the synonymy relation, but other things — such as occurrences of terms or classes of terms. Or perhaps we should not require concepts to be closed under the general relation of synonymy, but only under synonymy of unambiguous terms. But then we need an independent and formal account of ambiguity in order to state such a principle with any degree of precision. However, no such possible alternatives are considered in Bodenreider (2001), and indeed the issue of any puzzle involving concept closure under synonymy in the context of ambiguity seems to pass unnoticed.^6^

### 1.2 Methodology and the need for a more formal approach 

These two puzzles illustrate, if not a problem with the theory underlying the Metathesaurus, at least a continuing problem with its explication. I believe that, owing largely to the genealogy of the Metathesaurus from its origins in lexicography and thesaurus management, a primary source of the confusions surrounding its central concepts of term,synonymy and concept is that the Metathesaurus attempts to provide a theory of meaning without providing a theory of reference – always a dangerous enterprise. This is, perhaps, another way of putting some of the complaints found in Smith (2004) and Brenner (2002).^7^

Smith (2004, 2006) takes the Metathesaurus to task for its fuzzy account of concepts and one which seems to bind them tightly to linguistic entities such as terms without linking them in any obvious way to “reality”. His concern is that the Metathesaurus account of concepts (and related critical notions) at no point intersects with empirical reality: there is no link between the terms of the source vocabularies and things (objects, events, processes, etc.) in the world. He proposes several possible alternative views of concepts, ﬁnds each of them wanting, and proposes his own approach to “good ontology” and “good modeling” that is based on the notion of a universal rather than that of a concept. The advantage, he suggests, is that statements phrased in terms of universals “convey knowledge precisely because they represent relations between entities in reality”. While I will not attempt to deal here with Smith’s own proposal, I do have a certain degree of sympathy for it (at least in terms of its image painted in broad strokes). But two millennia of philosophy have demonstrated that a theory of universals (or natural kinds) is fraught with as many problems and challenges as is a theory of concepts. And in the absence of a precise and unequivocal account of concepts in the UMLS, it is unclear precisely in what way they are not universals.^8^

I am not convinced (as Smith seems to be) that the theory of meaning underlying the Metathesaurus is irredeemably mistaken and incoherent. I am even less convinced that a precise and scientiﬁcally meaningful approach to medical informatics requires a theory of universals to serve as its semantic base, and further that such a theory is to be found, as Smith proposes, in the work of D. M. Armstrong. (For an alternative view of an Armstrongian approach to universals, problems encountered with this, and some possible alternatives, see Lewis (1983, 1986a,b). And note that in Armstrong (1989), Armstrong himself concedes a nominalistic approach eschewing universals would address the same problems as does his own.) My point here is not that a universals-based approach may not be one way of solving some of the problems and confusions evident in the Metathesaurus, but that we cannot reasonably advocate for a universals-based alternative to the theory underlying the Metathesaurus – or for any other – if we do not know with some degree of precision what that theory is. 

The concept of synonymy appears to lie at the heart of the Metathesaurus, but it is never quite clear (as the examples of the previous section demonstrate) what ‘synonymy’ means. And in the empirical sciences, it is often less important that two terms are synonymous (mean the same thing) than that they refer to the same thing. But the Metathesaurus has a “dictionary orientation” which holds paramount relations of meaning or partial meaning. Concepts are then introduced in order to explicate – or model – meaning and synonymy among terms. They are (or at least often are purported to be) source-independent “universals” (though Smith would not agree with this characterization from the perspective of universals in classic meta­physics) that represent “meanings”. Still, in the Metathesaurus, synonymy historically has played a primary role. 

One rejoinder to Smith’s attack on concepts – compatible with a substantial portion of the literature on the UMLS^9^– would be to observe that indeed there is an intersection with empirical reality since the Metathesaurus rests solidly on how biomedical language is used in clinical and scientiﬁc contexts. I will return to a more detailed review of this argument in Sections 2.3 and 5.3. 

Smith would like to make universals the referents of general thesaurus terms. But there are alternatives to such an approach. However, it is not my goal to “repair” or “improve” the theory of meaning that is either explicit or implicit in how the Metathesaurus treats terms, concepts, and synonymy – nor particularly to defend this against criticisms that have been addressed to it. My goal is rather to propose a clear and unambiguous statement of this theory in order that we can determine with some precision what its features and consequences are, and to provide a framework within which statements and questions concerning the UMLS can be expressed with sufﬁcient precision that they may be compared and evaluated with objectivity. Thus I seek primarily to provide a comprehensible (though not necessarily comprehensive) framework that may be used as both a basis and a guide to understanding the UMLS; and this is as much an exercise in explication and pedagogy as it is in knowledge representation and semantics. 

### 1.3 Some fundamental questions 

As both a guide in constructing the desired framework and a means of judging its adequacy, we will begin by stating a series of questions about the Metathesaurus and its conceptual basis. These arise naturally from a consideration of the literature on the Metathesaurus and the sort of puzzles that arise from this: 

**Question 1**. What is a term? What, in the context of the Metathesaurus and explications of it, does ‘term’ mean? 

**Question 2**. What is the role of term types in characterizing and individuating terms? 

**Question 3**. What is the role of atoms in characterizing terms, concepts, and synonymy? 

**Question 4**. Can a term be ambiguous? What effect does this have on how concepts and synonymy can be characterized? 

**Question 5**. In what way are UMLS concepts not universals (in the sense of Smith (2004))? Can they be made to serve the role of universals? 

**Question 6**. Is the Metathesaurus representation of terms, concepts, and synonymy truly divorced from em­pirical reality, or is there a clear relation between the Metathesaurus and the empirical world of biomedicine? 

If in the end our formal theory allows us to answer these questions with precision, then we should count it as a success. 

## 2 Synonym

The characterizations of synonymy 10 found in UMLS 2 and UMLS 5 are essentially (and informally) these:

[Equivalence Principle of Synonymy]

t_1_ and t_2_ are synonymous just in case the substitution of (one or more occurrences of) one for the other in any sentence of our language yields an equivalent sentence.

The Equivalence Principle of Synonymy has the appearance of a deﬁnition of synonymy in terms of the substitutivity or interchangeability of terms in sentences. Indeed, UMLS 5 characterizes this as a deﬁnition, and historically it is an expression of the deﬁnition of synonymy as “saving truth” (salve veritate) under substitution. However, this is in itself a composition of two distinct principles:

[Interchangeability of Synonyms]

If t_1_ and t_2_ are synonymous, then any sentence S_1_ is equivalent to a sentence S_2_ that is identical to S_1_ except for containing an occurrence of t_2_ where S_1_ contains an occurrence of t_1_.

[Synonymy of Interchangeables]

If all pairs of sentences S_1_ and S_2_ – which differ only in that S_2_ contains an occurrence of t_2_ where S_1_ contains an occurrence of t_1_ – are equivalent, then t_1_ and t_2_ are synonymous.

The ﬁrst of these describes a consequence of synonymy: that the two terms can be everywhere inter­changed; and the second describes a criterion of synonymy: that terms are synonymous if they are every­where interchangeable. Keeping these principles distinct is key to understanding the role of synonymy in the Metathesaurus. And if we view them separately, then – rather than thinking in terms of a deﬁnition of synonymy – we may view each as a proposed constraint on a semantic theory of concepts and synonyms. This is an important conceptual step for two reasons. 

First, the well-known discussions of Mates (1950) and Quine (1980b,c) provide detailed accounts of why the Equivalence Principle cannot be considered as an adequate deﬁnition of the concept of synonymy. They also provide accounts of how the Interchangeability of Synonyms and the Synonymy of Interchangeables are individually ﬂawed and in general hold only in restricted ways. We will not reexamine these arguments here, but a consequence is that it would be a fundamental error to take the notion of synonymy as characterized by the Equivalence Principle as the foundation of a theory of meaning or concepts as desired for the Metathesaurus. 

Second, once we have split the Equivalence Principle into these two separate principles, it becomes possible to consider accepting one of them while rejecting the other. And this is the path we shall take as our formalization of these notions progresses: I shall accept something akin to the Interchangeability of Syn­onyms, providing us with a powerful concept-based inferential mechanism within the Metathesaurus. This will also expose some confusions and dangers in focusing on synonymy as the fundamental semantic rela­tion supporting the construction of concepts. But I will reject any form of the Synonymy of Interchangeables as being both unnecessary and incompatible with the manner in which the Metathesaurus is constructed, extended, and maintained. 

A consequence of this approach is that I shall view synonymy as a primitive semantic relation within the Metathesaurus – related to truth, meaning, and semantic equivalence, but not deﬁnable in terms of these.^11^And I shall argue that reliance on it in either a formal or explicatory role within the Metathesaurus is misleading and problematic. 

### 2.1 Synonymy, terms, and atoms 

In descriptions and discussions of the Metathesaurus (and in medical informatics more generally) the term ‘synonymy’ often refers, not to true synonymy (strict sameness of meaning), but to some weaker relation of quasi-synonymy or translation. This is most explicitly pointed out in 

UMLS 10

“However, synonymy in practice is fuzzier. Meaning may have multiple aspects. In the search for synonyms, it is rare to ﬁnd two names having identical meanings. It is more common to ﬁnd names that overlap closely in meaning but are not equivalent in all situations (plesionymy). Thus, between true synonymy and nonsynonymy, there may be “relative synonyms,” which even though they do not satisfy the strictest deﬁnition of true synonymy, are close enough in meaning to be considered “practically synonymous” in certain circumstances. In controlled vocabularies, relative synonyms may be treated as if they named exactly the same concept.”

Fung, et al. (2005)

and is compatible with the view taken in the Metathesaurus concerning the potential ambiguity of terms as well as the distinction between “local” synonymy within a source vocabulary contrasted with a “source transcendant” sense of synonymy that supports the construction of concepts as this is described in McCray and Nelson (1995), Bodenreider, et al. (1998), Hole, et al. (2004), and in Fung, et al. (2005). 

These observations can also be taken as recognition that the arguments found in  Mates (1950) and  Quine (1980b,c) compel the view that any coherent sense of synonymy must be one in which the relation is taken to be context-dependent in some way. Mates and Quine urge the view that synonymy makes sense only when relativized to a language, and that (depending on the nature of the language) strict synonymy may require more than interchange salva veritate. Such considerations also raise the question of whether there is in fact any coherent transcendant sense of synonymy in which two terms found in distinct source vocabularies of the Metathesaurus can be said to be synonymous in other than a vague and imprecise manner. Yet it appears that the very idea of source-transcendant concepts in the Metathesaurus depends upon there being such a source-transcendant sense of synonymy – since, according to most of the literature and documentation pertaining to the Metathesaurus, concepts are just collections of (transcendentally) synonymous terms. This appears in turn to force us to the view that if there is any sensible transcendant sense of synonymy, it cannot be one in which the synonymy relation obtains among terms. At this point, a simple non-biomedical example may help – and may aid in exposing even more dimensions of the problem. 

Suppose you go to a restaurant, sit at a table, and wish to order dinner. Almost certainly, the waiter will offer you a menu. But suppose he doesn’t (or suppose you’ve sat at the bar and want to order dinner). What do you ask for and how do you ask for it? Obviously, you say "I would like a menu." Now suppose that this is a French restaurant in, say, Paris. Then you need to ask for a menu in French. If you want a menu (English), then you commonly would ask for a carte (French): “La carte, s’il vous plait.” So we might say that “In French, ‘carte’ means what ‘menu’ does in English” or “The word ‘carte’ in French is synonymous with the word ‘menu’ in English.” But while the ﬁrst of these statements is true in some sense (in one sense of ‘carte’), the second is hardly true at all. At best, it becomes true when expanded to something like “The word ‘carte’ in French is synonymous with the word ‘menu’ in English when used in a restaurant.” And even this isn’t quite true, for how would you ask for a map in the restaurant? (Certainly in that context you would need to be a bit more speciﬁc – “Je voudrais une carte de Paris.” – but you would use the French word ‘carte’ and not mean ‘menu’.) The moral of this story is, ﬁrst, when we say that the French word ‘carte’ is a synonym of the English word ‘menu’, this is correct only when a certain set of constraints (or a speciﬁed context) is imposed on it; and, second, that it is not words (or terms) that may be synonymous with one another, but rather occurrences of words (or terms) within speciﬁc contexts. And any account of synonymy, or meaning, or sameness of meaning must accommodate this fact. Interestingly, this example extends to the Metathesaurus since within it the term ‘map’ is also ambiguous across multiple sources. 

A rather direct result is that assertions such as those found in UMLS 1 -UMLS 6 attributing synonymy to terms (or names) are – strictly speaking – mistaken. At the very least, they are misleading and confusing. That synonymy in the Metathesaurus is not a (direct) relation among terms is acknowledged explicitly in Fung, et al. (2005): 

UMLS 11

“An atom in UMLS is a unit of meaning from the source vocabulary. A UMLS concept is made up of one or (usually) more atoms ... In other words, all atoms having the same CUI are synonymous in the UMLS view.”

Fung, et al. (2005)

Indeed, this appears to be the only UMLS publication in which the critical point is made that it is atoms (oc­currences of terms) that are synonymous with one another and atoms that are the constituents of concepts. A coherent view of synonymy in the Metathesaurus requires the recognition that synonymy is not a relation among terms – or at least that it is fundamentally a relation among atoms and only perhaps derivatively a relation among terms. We will explore this issue more fully and formally in Section 4.4.2 below. 

### 2.2 Synonymy and ambiguity 

The example of ‘menu’, ‘carte’, and ‘map’ additionally illustrates the problems encountered in thinking of synonymy as applied to ambiguous terms. The claim that two ambiguous terms are synonymous can make sense only if they are ambiguous in exactly the same way in exactly the same contexts – so that their ambiguous meanings “match” (*cf.*  Mates (1950)) – a very unusual occurrence. This again enforces the view that it is not terms (stripped of context) that should be considered as synonymous, but terms under a particular “interpretation” of them that associates with each term a particular “sense” – which is to say disambiguated terms or disambiguated term occurrences. In turn, this should lead us to realize that such a notion of sense or meaning is therefore more fundamental than that of synonymy, which is a direct derivative of it. Such a realization is reﬂected in the ﬁrst sentence of UMLS 11, but it is often lost sight of in published discussions of the Metathesaurus. 

We shall return to these points as the development of our formal approach progresses. 

### 2.3 The determination of synonymy 

An important question we have not yet addressed is “How is synonymy determined?”. That is, given two terms t_1_ and t_2_ (or atoms a_1_ and a_2_), how do we tell whether t_1_ (a_1_) is in fact synonymous with t_2_ (a_2_)? We will address this question now in an informal manner – at times blurring, for the sake of readability, the distinction between terms and atoms that we have just been so careful to introduce. Later, we shall reconsider it in a fully formal context. 

The principle of the Synonymy of Interchangeables, as we have already mentioned, provides us with a criterion of synonymy. So it would appear that we need only apply it in order to determine the sets of synonyms in our language. How do we do this? 

Well, we could begin by listing all the sentences of our language (there will typically be a countable inﬁnity of these). To make this easier, we might restrict this approach to listing only the “simple” sentences of the language (without logical connectives, complex phrases and sub-phrases, etc.). There will likely still be countably many. At the very least there will be a great number of them. Then we could iterate repeatedly through this list, determining which of these sentences are “equivalent” to which others and extracting the pairs of terms (identiﬁed positionally) that occur in the pairs of equivalent sentences. This should take only a countably inﬁnite amount of time. Even with a fast computer, that is a long time. At this point it begins to appear that while the Synonymy of Interchangeables may provide a criterion of synonymy, it is not an especially useful one. Moreover, this is not how synonymy is in fact determined – particularly with respect to the Metathesaurus. 

In practice, whether two terms are synonyms is determined – not by a consideration of interchangeabil­ity in all the sentences in which they may appear – but by competent language speakers reﬂecting on the meanings of the terms and deciding whether in fact they mean the same thing. In the case of the Metathe­saurus, synonymy is determined in much the same way, and this is described in detail in Fung, et al. (2005) and in other examples such as McCray and Nelson (1995) (see UMLS 7), and in Section 2.2 of NLM (2008): 

UMLS 12

“The construction of the Metathesaurus is based on the assumption that specially trained subject experts can determine synonymy with a high degree of accuracy. Metathesaurus editors decide what view of synonymy to represent in the Metathesaurus concept structure.”

NLM (2008)

The process of adding a new source vocabulary to the Metathesaurus involves two steps (Fung, et al. (2005)): 

**Automated alignment of terms.** A set of algorithms and heuristics are used to “match” the terms of the new vocabulary to concepts (which recall are construed as sets of synonyms) already in the Metathesaurus. 

**Manual review and determination.** UMLS editors review the results and make any changes necessary in order to bring them into conformance with their (shared) expert knowledge of the language use. 

There are three important features of this process that bear emphasis. First, the initial step – which ap­pears to be one involving no “manual” component – in fact depends heavily on such a component since the individual sources previously integrated into the Metathesaurus all resulted from processes of manual construction, editing, and curation. Second, the keepers of the Metathesaurus do not determine that two terms are synonymous because they share the same concept. Rather, they determine that terms (actually, term occurrences) share a concept because they are synonymous. Synonymy, rather than meaning, is treated as the fundamental semantic property. And third, the relation of synonymy in the Metathesaurus is ultimately determined by the agreement of competent language users (i.e., users of biomedical language and of the individual source vocabularies comprising the Metathesaurus). Ultimately, two terms are deemed to be synonymous because that is how they are used by the community of language users. 

### 2.4 A summary of synonymy 

This review of the nature and role of synonymy in the Metathesaurus has led us to some important con­clusions, at least some of which may seem surprising from the perspective of our original view of the Metathesaurus through the lens of UMLS 1 -UMLS 8:

Synonymy does not apply to terms, but to occurrences of terms in particular languages (in the Metathesaurus, to atoms in source vocabularies).
As a consequence, the coherence of any source-transcendant sense of synonymy is in question.
Such principles as the Equivalence Principle of Synonymy, the Interchangeability of Synonyms, and the Synonymy of Interchangeables must be understood to apply to occurrences of terms (atoms) rather than to terms themselves.
Synonymy in the presence of ambiguity requires disambiguation: there is no clear fundamental sense in which we can meaningfully speak of the synonymy of ambiguous terms, though we can meaningfully speak of the synonymy of disambiguated occurrences of terms.
In the Metathesaurus, synonymy functions as a primitive semantic relation that is fully determined by a community of competent language users (including Metathesaurus editors and editors or curators of the individual source vocabularies).

We can also claim to have taken some signiﬁcant steps in answering questions 1-4 of Section 1.3; and with this understanding of synonymy in hand, we are prepared to take the next step in pursuing a more formal analysis of these ideas. 

## 3 Logical and linguistic preliminaries 

In general, I take NLM (2008) to be the deﬁnitive characterization of the UMLS Metathesaurus and take other publications to be explicatory with respect to it. On this basis we can identify the fundamental elements of the Metathesaurus as those appearing in Table 1.

**As a characterization of meaning, semantics (or a semantics) requires three constituents:**
A language. The language is the carrier of meaning – or more properly, it is expressions in the language that are meaningful.

**Table 1:**
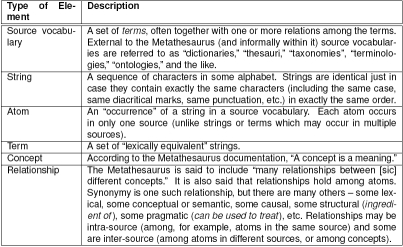
Formal Elements of the UMLS Metathesaurus

Something that the language is about or that it expresses. This is sometimes referred to as the domain.
A description of the relation between the language and what it is about or expresses. This description is then the speciﬁcation of the concept of meaning for the language relative to its domain.

This brief characterization of semantics allows a semantics to be a relation between a language and a non-linguistic domain (the, or a, world); or it allows a semantics to be a relation between two languages (or perhaps even a language and itself). 

In the case of the Metathesaurus, we may think of each source vocabulary as one possible view, descrip­tion, or representation of reality. MedDRA is one possible description of (roughly) the domain of medical conditions, while ICD-9 CM is another;^12^and GO (the Gene Ontology) is one possible description of the domain of biological processes, molecular functions, and cellular components. Among these possible de­scriptions, some are broader than others and there are degrees of overlap and of granularity. SNOMED-CT is both broader (it is a description of drugs as well as of medical conditions), more granular (it generally recognizes more distinctions among kinds of things) than MedDRA or ICD-9 CM, and more complex. Yet at the same time ICD-9 CM, for example, contains some distinctions and some terms not represented in SNOMED-CT. The relations among these sources – as possible descriptions – are complex. Each can be thought of as a partial description of a portion of reality (its “intended domain”), comparable in certain clear ways to other such partial descriptions, but not comparable in others. 

We must not lose sight of the fact that the goal of the Metathesaurus is that it be applied in a broad variety of empirical (clinical, research, and informatics) contexts. It is natural, then, to think of the terms of a source vocabulary appearing in the statements of what may be thought of as an application language in which descriptions of the empirical biomedical domain may be formulated and employed in inferences. Indeed, understanding the role and description of synonymy in the Metathesaurus requires reference to such a language in which statements may be made and in which the truth and falsity of such statements may be assessed. From an informal point of view (and from the perspective of typical thesaurus characterizations as found, for example, in MedDRA (2007), NISO (2005), and NLM (2008)), the application language is most often taken to be a variety of “technical English” (or some other natural language) in which such assertions as

Ibuprofen may be used to treat inﬂammation.
If an A1C test result is above 5%, then diabetes is indicated.

can be made – and where in clinical and scientiﬁc settings, conditions or symptoms (as represented in, say, ICD-9 CM or MedDRA) can be expected to be related to procedures (as represented in CPT) or drugs (as represented in RxNorm or its constituent sources). Thus while each of its source vocabularies is intended to apply independently to a particular domain, in practice terms from multiple Metathesaurus vocabularies will appear in statements, assertions, hypotheses, queries, and inferences that relate entities from one domain to entities from others. And while source vocabularies are distinct from one another in the Metathesaurus (with, as we have observed, potential overlap of terms), the Metathesaurus informally recognizes a broader language and set of relationships in which these terms appear and which transcend the set of vocabularies. Examples of such relationships include active_ingredient_of,associated_disease, cause_of, ingredient_of, metabolizes, may_prevent, etc. While in the data model of the Metathesaurus these appear as “relationship attributes”, we shall treat them here as relations represented by relation expressions such as ‘is an active ingredient of’, ‘is an associated disease of’, ‘is a cause of’, etc. Such relations may obtain both within a single source and across multiple sources.^13^

The view that emerges from these considerations is then that each source in the Metathesaurus is a vocabulary of terms, that such terms may appear in one or more application languages (which describe data or relations in the empirical world), and that a thesaurus may be used in conjunction with such an application language to achieve certain results pertaining to information or knowledge represented in the application language. However, expressed in this informal manner, such a view is quite vague and gives rise to a number of questions. 

A thesaurus is a collection of terms - but what, exactly, is a term?  And how do thesaurus terms function– syntactically, semantically, and pragmatically – in a broader linguistic or logical context? And how do thesauri function in their applications within the empirical sciences? The answers to these questions are important, particularly if we want to understand how thesauri may be applied to the empirical world, how they may be employed to enhance knowledge acquisition and inferencing, and how this may be done within a variety of formalisms and formal methodologies that include logic, machine learning, artiﬁcial intelligence, and statistical inference. 

### 3.1 Purposes and roles of formal thesauri 

Documents that describe particular thesauri, and documents that more generally characterize the nature and structure of thesauri, traditionally avoid addressing these questions in other than a cursory manner while assuming that the answers to them are sufﬁciently straightforward or obvious to provide the reader with an understanding of how the thesaurus may be applied. The underlying assumption pertaining to thesaurus application is that this is accomplished by making use of the thesaurus terms and relations within a richer language – the precise nature of which is left vague, but which is most often assumed to be “natural language” or some variant of that. This approach is almost certainly an artifact of the history of what might loosely be referred to as “thesaurus theory” in the ﬁelds of library science and, more recently, informatics. From this perspective the primary (if not the only) roles of a thesaurus lie in (a) determining (as a look-up mechanism) precisely what term ought to be used for a particular concept within a speciﬁc context (e.g., a legal context, a marketing context, a context involving patent ﬁlings, or a regulatory context), (b) aiding in information retrieval – typically at the document level – by providing a set of search terms (related to a term of interest) that can be used in query expansion to achieve “concept enhanced queries”, and (c) the classiﬁcation or categorization of documents for the purposes of information organization or knowledge management and to enhance capabilities of searching and browsing documents. For the sake of brevity, we can refer to these thesaurus roles, respectively, as the usage role, the *information retrieval role*, and the *classiﬁcation role*. 

In each of these classic thesaurus roles, the target of thesaurus application is a document (or a corpus of documents) where a document is simply a set of sentences in natural language. It is documents (or perhaps sub-documents), then, that are classiﬁed or retrieved; and the role of the thesaurus is to aid in such retrieval or classiﬁcation by providing a semantic context in which queries or classiﬁcation criteria may be enhanced to achieve better or more complete results. In the case of the usage role, a document is transformed into a different document through the application of *preferred term*,*use*, or *use-for* relations that results in a largely equivalent document that may be thought of as “more proper” or as conforming to certain constraints concerning the “best” or “most acceptable” use of terms. 

In these historical contexts of thesaurus use, the scenario is that there is a corpus of documents in natural language, that the thesaurus consists of “terms” drawn from that natural language, that each term “represents a concept” or “designates a concept”, and that otherwise ‘term’ is equivalent to ‘word’, ‘one or more words’, or ‘phrase’. The further (often tacit) assumption is that the terms occur in the thesaurus just as they occur in the document corpus – since, after all, the terms were extracted into the thesaurus from natural language itself. 

More recently, some additional – and highly signiﬁcant – roles for thesauri have emerged: the knowledge discovery role, and the inferential role. An understanding of these makes it necessary for us to reconsider the classic scenario concerning thesauri, their terms, and their application to document corpora. 

### 3.2 The languages of a thesaurus and its application 

If we turn turn our attention from such questions as “How can we ﬁnd all the documents whose content is relevant to concept C?” (which is an information retrieval question) and “How may we assign each document in our corpus to one or more categories?” (which is an information classiﬁcation question) to such questions as “What do we know about concept C (or objects realizing concept C) based on evidence E?” or “What can we infer about concept C (or about objects realizing concept C) based on our knowledge set K?”, then the relation of a thesaurus to answering these questions becomes a bit more complex. Consider, for example, the difference between addressing the question “Into what categories should each of the papers in the 2008 issues of the *Journal of the American Medical Informatics Association* be classiﬁed?” and addressing the question “On the basis of the information contained in the Acme Insurance Company’s prescription claims data base, what drugs are related to an increased risk of myocardial infarction?”. 

The question concerning the classiﬁcation of papers in *JAMIA* views the information at issue at the granularity of documents. And the classiﬁcation problem can be addressed in well known ways that involve the application of a variety of statistical methods and models to documents viewed as sequences of words, sentences, and paragraphs. See, for example, the chapters on clustering, information retrieval, and text categorization in Manning (1999). The methods employed certainly may yield improved results if a the­saurus is used to enhance their application (so that, for example, synonyms are recognized as representing the same concept or that narrower terms are recognized as representing the concepts of their broader terms). But this example then falls ﬁrmly within the classic classiﬁcation role of thesauri and conforms to the assumption that the language of the thesaurus and the language of its application target do not differ in signiﬁcant ways. Both are English or fragments of English; and moreover, the structure of the text or information in the documents being categorized is of little or no signiﬁcance since the primary goal is to identify only the presence (or perhaps prevalence) of a relevant concept within a document rather than to identify relations among entities that realize concepts of interest. The type of question being answered is “Is this document about aspirin or not?” rather than “How is aspirin related to blood pressure?”. 

On the other hand, our question about an increased risk of myocardial infarction (and similarly our question about aspirin and blood pressure) – and whether this is supported by information in a particular data base – is a wholly different sort of question since it is a question about whether a particular statement is true (and further, whether it is true on the basis of certain evidence). In the case of knowledge discovery and inference, the application domain is not viewed simply as a set of documents but as an objective empirical domain that is described and represented in a language within which inferences may be made. Thus in terms of the knowledge discovery and inferential roles of thesauri, the objects of analysis are not documents but rather statements (or sentences, or assertions) construed as being  *about* a particular empirical domain. An application language in which truth-bearing expressions such as sentences, statements, or assertions can be formed is necessary in order to even state such characterizations of synonymy as are found in UMLS 2 and UMLS 5. In providing a formal model of thesauri and their application to empirical domains, it is therefore essential to be clear about the language of the thesaurus, the language being used to describe the empirical domain (and to make inferences), and the relation between these.

### 3.3 When is a term not a term? 

In linguistics, a term is normally taken to be a noun or noun phrase. In formal logic, the terms of a language are typically deﬁned to include variables, constants, the result of applying an n-place operation symbol to n terms, and descriptive terms (‘the thing x such that ...’). In each of these cases, what is at the heart of being a term is that the term purports to denote (or refer to, or stand for) some thing. Suppes (1999) provides such a semantic characterization of terms by saying 

Such a usage of ‘term’ is also compatible with terms as these appear in ancient and medieval logic, as they appear in description logic, and with the treatment of terms in NISO (2005) where it is said that a term is “one or more words used to represent a concept”. 

Among terms, it is then common to distinguish between *singular terms* and *general terms*. A singular term denotes – as its name suggests – a single individual entity. A general term is then taken to denote (severally) a number of individual entities, a class of such entities, or perhaps a property of such entities. In philosophy it has been very popular at times and in various circles to hold that a general term denotes a *universal* (or for Plato, a *form* or *idea*). In the classic example, “Socrates is a man,” the term ‘Socrates’ is singular and denotes a particular man while the term ‘man’ may be taken to be a general term denoting the class of men, the form *Man*, or mankind. Alternatively, in modern ﬁrst-order logic, ‘man’ would not be regarded as a term in this sentence, but rather ‘is a man’ would be regarded as a predicate. And the difference here is that predicates are not (or certainly *need* not be) viewed as *denoting* anything. Rather, in the context of a sentence, a predicate is used to assert something about an individual (or to assert a relation between or among individuals) – without the presumption that the predicate denotes any entity in the world. 

I belabor this well-worn logical and grammatical point because insufﬁcient attention to it can result in some degree of confusion when applying thesauri to certain familiar kinds of application languages. The­sauri, again, are sets of terms. And standards such as NISO (2005) recognize different types of terms that may appear in thesauri, but these are all either genuine terms (in the sense we have been discussing) or are construed as “nominalizations” of other parts of speech (see NISO (2005), section 6.4). However, a particular formal application language may not contain, for example, general terms – representing such concepts instead as predicates: ‘is an instance of Hodgkins Disease’ rather than ‘Hodgkins Disease’. And so the general terms in a thesaurus being applied through such a language would have as their counter­parts predicates rather than terms. That is, the terms of the thesaurus would not be found in the application language (at least as terms). The more general point here is that the grammatical categories and syntax of the thesaurus may differ signiﬁcantly from those of the application language, and any formalization of thesauri and their applications must accommodate this fact. 

### 3.4 Characteristics of an application language 

In order to make use of a terminology or vocabulary, the terms of the terminology must appear in a language in which assertions can be made. Once this is done, it is then possible to speak meaningfully of the truth or falsity of such assertions, and hence also of such principles as the *Interchangeability of Synonyms* and the *Synonymy of Interchangeables*. In addition, as a practical matter a thesaurus may be used only by means of its terms appearing, in some way or other, in an application language. 

An application language may take one of many forms. It may, for example, be one or another data query language such as SQL, it may be the language of a description logic, it may be a knowledge representation language, or it may be a logical language such as standard ﬁrst-order predicate logic (possibly extended with a theory of sets). But it is the application language that allows us to make statements, formulate queries, or represent relations among the things for which thesaurus terms may stand. 

For our purposes of viewing the UMLS Metathesaurus from a model-theoretic perspective, it will be instructive to abstract and identify those features of an application language that are essential to its use in the knowledge discovery and inferential roles of thesauri in exploring and analyzing biomedical data. Accordingly, we take an *application language 𝔏 for a thesaurus 𝔗 *as any language satisfying the conditions:

𝔏  contains a set of expressions which are *truth-bearers*. Such expressions are typically referred to as the sentences of  𝔏. To say that they are truth-bearers is to say that such an expression is either True or False. It is by means of such truth-bearers that statements (about a given domain or domains) are expressed. 14
𝔏  contains a set of expressions occurring in (but distinct from) the truth-bearers of  𝔏 which correspond to (or express) the terms in 𝔗. We will refer to this set of expressions as the *term-surrogates of 𝔗 in  𝔏 *.

Note, again, that the term-surrogates need not be terms (i.e., expressions that are thought of as *denoting*). That is, we take our thesaurus 𝔗 to be a set of terms in Suppes’ sense which are assumed to *name* or *denote* particular objects. But the surrogate in our application language  𝔏 of such a term may not be a denoting expression (a term of  𝔏 ), but rather a predicate, verb, or other expression whose semantics does not require that it denote. For example, ‘bacterial infection’ may be a *term* in the thesaurus 𝔗, but may be represented by the *predicate* ‘is a bacterial infection’ or ‘is an instance of a bacterial infection’ in the application language  𝔏. The point here is that the components of an application language may differ in fundamental ways from the components of a thesaurus – which are simply thought of as terms. And indeed, typically an application language will differ in these ways because it requires a substantially richer expressive power than can be provided by a collection of terms. In addition,

A relation that associates with each truth-bearer in  𝔏 one of the truth-values True or False is called a *truth-value* interpretation of  𝔏.
A function r that assigns to each term τ in 𝔗 exactly one term-surrogate r(τ) in  𝔏 is called a *term representation* of 𝔗 in  𝔏 .
If S is a sentence of  𝔏 and r is a term representation of 𝔗 in  𝔏 , then S^i^_r,τ,η_ is the result of replacing the *i*-th occurrence of r(τ) in S with an occurrence of r(η). That is, S^i^_r,τ,η_ is the result of replacing the i-th occurrence of τ’s surrogate in S with an occurrence of η’s surrogate.

Thus an application language for a thesaurus is a language containing sentences that are either true or false and which may contain the terms of the thesaurus (or at least their surrogates under translation as described in the previous section). 

We assume that an *application language* allows us to model simple subject/predicate or relational sen­tences such as “Lipitor is an HMG-CoA reductase inhibitor” or “Smoking is related to lung cancer” as well, perhaps, as (Boolean) sentential compounds and perhaps more complex constructions. More generally, this notion can be extended to that of an application language for a set of thesauri. And having such a for­mal (or at least quasi-formal) characterization of application language will then allow us to formulate such principles as the *Interchangeability of Synonyms* in a precise and formal manner. 

By way of a brief example, we may consider adopting an application language based on standard ﬁrst-order predicate logic – what we might call a ﬁrst-order application language. Here the notions of *term*, *predicate*, and *formula* (or *sentence*) are rigorously deﬁned, and a truth-value semantics may be provided that makes it possible to speak of the truth values of sentences without becoming entangled in various issues of denotation, extension, and the interpretation of general terms.^15^

Given a thesaurus 𝔗, it is then possible to deﬁne a term representation of 𝔗 in this language. Having thus characterized what a ﬁrst-order application language is, however, it is at this point not clear exactly what this term representation relation should be. In fact, there are two possibilities: a thesaurus term might appear as a *term* of the application language  𝔏, or it might appear as a predicate of  𝔏. In the ﬁrst case, a statement such as “Lipitor is an HMG-CoA reductase inhibitor” would be rendered as a relation between two terms (‘Lipitor’ and ‘HMG-CoA reductase inhibitor’) and this relation is the binary is-a relation. We may refer to this as the *term representation of general thesaurus terms in (the application language)*  𝔏. But in the second case it would be rendered as a universal statement where both ‘is (an instance of) Lipitor’ and ‘is (an instance of an) HMG-CoA reductase inhibitor’ are taken to be 1-place *predicates*, the statement is rendered as ‘Every instance of Lipitor is an instance of an HMG-CoA reductase inhibitor’, and neither ‘Lipitor’ nor ‘HMG-CoA reductase inhibitor’ appears as a term of  𝔏. We may refer to this as the *predicate representation of general thesaurus terms in*  𝔏. 

If our application language had the structure of a class logic (which recognized classes or sets and terms denoting them), or if, for example, it was the language of a description logic, then the term representation would be appropriate and more natural. However, for an application language based on standard ﬁrst-order logic, the predicate representation is more natural so that reasoning could be done based on standard ﬁrst-order rules of inference. 

Throughout the ensuing discussion and more formal development, we will assume – without developing the details – that the notion of an application language is clear, that we can meaningfully speak of the truth and falsity of sentences in such a language, and that the uniform representation of thesaurus terms and the operation of replacing (the surrogate of) one thesaurus term with another in sentences of the language can be given formal precision. 

## 4 Steps towards a formal semantics 

We may think of an application language for the Metathesaurus as related to a particular source (a “source application language”), or we may think of there being a broader application language that spans the in­dividual source application languages and allows us to formulate assertions containing terms from these various sub-languages. This second view is necessary if we are to make use of the Metathesaurus as it is intended. 

### 4.1 Metathesaurus frames and application languages 

How then are we to represent this application language and its relation to thesauri that comprise the Metathesaurus? We begin by attempting to model the fundamental aspects of a UMLS Metathesaurus source vocabulary. 

**Deﬁnition 1**. A Metathesaurus vocabulary frame 𝔉  is a sequence (V,O, A) where 

(1)  V (the vocabulary of 𝔉 ) is a non-empty ﬁnite set of strings. 

(2) O (the occurrences of terms in 𝔉 ) is a non-empty ﬁnite set. 

(3)  A is a function whose domain is V and  

  (a) for each string τ ε V, A(τ) is a non-empty subset of O 

  (b) if A(τ) ∩A(η) is non-empty, then τ = η 

We will often refer to Metathesaurus vocabulary frames more succinctly as *vocabulary frames* or simply as *frames*. If 𝔉 = (V, O, A) is a frame, then we will also refer to O as the *atoms* (or *set of atoms*) of 𝔉  and to A(τ) as the *atoms* of (the term) τ in 𝔉 . In the absence of specifying V, O, and A explicitly for a frame 𝔉  we will employ the notational convention of referring to these as V_𝔉_, O_𝔉_, and A_𝔉_. 

Clause 3 of Deﬁnition 1 ensures that (a) every term in the vocabulary of a frame has at least one occurrence (atom) in that frame, and (b) that no two distinct terms may share an occurrence (atom). The latter condition will contribute to the effect that within a frame, each atom is associated with a distinct “sense” and role (or use) of a particular term.  16

**Deﬁnition 2**. If 𝔉  is a Metathesaurus vocabulary frame, then α exempliﬁes τ in 𝔉  (or α is an exemplar of τ in 𝔉) if and only if α ε A_𝔉_(τ).

Informally: An atom exempliﬁes any term of which it is an atom. (Hence A_𝔉_(τ) is the set of exemplars of τ in 𝔉 .)

It is tempting to think of each Metathesaurus atom as representing, within a particular vocabulary, a distinct sense or meaning of the term it exempliﬁes. Something like this seems to be suggested in UMLS 11 where it is said that “An atom in UMLS is a unit of meaning from a source vocabulary.” However, this is, at the very least, quite misleading if not outrightly incorrect. 

The Metathesaurus documentation deﬁnes an atom as “an occurrence of a string in a source vocabu­lary,” but unfortunately it does not deﬁne ‘occurrence’ nor indicate the precise circumstances in which one occurrence differs from another. Examples in NLM (2008) encourage the view that each such occurrence corresponds to a particular sense of the atom’s term, but in fact this is not the case and two distinct atoms may have exactly the same sense or meaning within a given source vocabulary. A clear example of this is found in the MedDRA source where, for each entry having a preferred term (PT), there are two distinct atoms: one for the PT “form” of the term and one for the LT (lowest level term) “form” of the term – even though this is the same term (i.e., is exactly the same lexical form – the same character sequence)! Thus this is a case where we have the same term with the same sense, but with distinct atoms distinguished by how the term is viewed (as an LT or its “equivalent” PT) or what its type or role is in the source vocabulary. (For an extended discussion of the LT/PT relation in MedDRA, see Merrill (2008b).) An atom, then, is not simply a lexical entity (sequence of characters), nor a lexical/semantic entity (sequence of characters with a particular sense or meaning), but rather it is a lexical/semantic/pragmatic entity (sequence of characters with a particular sense and a particular role or use). Indeed, the precise nature of atoms in the Metathe­saurus is shrouded in mystery, not documented with any degree of completeness or precision, and buried in unpublished UMLS program code that is employed to generate and distinguish atoms when a source vo­cabulary is imported into the Metathesaurus. This makes discussing the precise role of atoms in the UMLS 

– and their relations to terms, concepts, and synonymy – difﬁcult and frustrating. However, for our purposes it should be sufﬁcient to think of an atom as a combination of a term, a semantics (sense or meaning) for that term, and a pragmatics (role or use) for that term:

atom = term(string) + sense + use.

Hence, as a reasonable and practical approximation, an atom can be thought of as a *(string, sense, use)* triple, and we may take the view that two atoms are identical if they are characterized by the same string, same sense, and same use.^17^A vocabulary frame captures the simple idea that a source is comprised of a vocabulary of strings (terms), each of which has one or more occurrences in the vocabulary, represented as atoms. 

Our characterization of application language provides us only with a formal characterization of such languages and in no way links these to the Metathesaurus. To accomplish this, we need the further notions of a *Metathesaurus frame set* and of a *representation* of a frame set in an application language. 

**Deﬁnition 3**. 𝔉^∗^ is a Metathesaurus frame set if and only if 

(1)  Every member of 𝔉^∗^ is a Metathesaurus frame. 

(2) For every 𝔉 _1_ = (V_1_, O_1_,A_1_) and 𝔉 _2_ = (V_2_, O_2_, A_2_) in 𝔉 *, if O_1_ ∩ O_2_ is non-empty then F_1_ =𝔉 _2_. 

*Informally*: A Metathesaurus frame set is a set of frames in which no two share an atom (occurrence of a term). 

This deﬁnition enforces the Metathesaurus constraint that an atom occurs in only one source, and we can take a frame set to represent a set of distinct Metathesaurus source vocabularies. 

It will also be convenient to speak of the atoms of a frame set, which simply comprise the set of all atoms of any of the frames in the set, the terms of a frame set, which similarly comprise the set of all terms of any of the frames in the set, and the relation of exempliﬁcation extended to a frame set: 

**Deﬁnition 4**. If 𝔉^∗^ is a Metathesaurus frame set, then 

(1) Atoms(𝔉^∗^) = ∪{O : ∃V∃A (V, O,A)ε 𝔉^∗^}. Informally: The set of atoms of the frame set 𝔉^∗^ . 

(2) Terms(𝔉^∗^) = ∪{V : ∃O∃A (V, O,A)ε 𝔉^∗^}. Informally: The set of terms of the frame set 𝔉^∗^ . 

 (3) α exempliﬁes τ in 𝔉^∗^ just in case α exempliﬁes τ in some 𝔉  ε 𝔉^∗^ . 

*Informally*: Informally: An atom exempliﬁes a term in a frame set just in case it exempliﬁes the term in some frame of the set.

Note, however, that although atoms (occurrences of terms) are not shared among frames in a frame set, the terms (strings) themselves may be. This means that while an atom exempliﬁes only one term, a term may be exempliﬁed by multiple atoms. In fact, a term may be exempliﬁed by multiple atoms in the same source (frame). A simple example of this occurs in MedDRA where an atom for a Preferred Term and a (distinct) atom for its corresponding Lowest Level Term both exemplify the same term. 

In practice, many terms may be shared among multiple source vocabularies of the Metathesaurus – as in the case of ICD-9 CM, ICD-10, Clinical Codes V3, MedDRA, and SNOMED-CT which all contain tens of thousands of terms pertaining to medical conditions. A frame represents a single source vocabulary through its fundamental elements: terms (strings), and occurrences of terms (atoms). And each Metathe­saurus release can be thought of as comprising (among other components) a frame set which collectively represents its source vocabularies. 

**Deﬁnition 5**. If 𝔉^∗^ is a Metathesaurus frame set and L is an application language, then a term representation of 𝔉^∗^ in L is a function r where: 

     (1) Domain(r) ⊆ ∪{V : ∃O∃A (V, O, A)ε 𝔉^∗^}. 

  (2)  For each τ in the domain of r, there is exactly one expression ξ of L such that r(τ)= ξ. 

*Informally*: A term representation of a frame set assigns to a term in the vocabulary of the frame set a single surrogate expression in the application language 𝔏. Thus a term representation “embeds” a set of source vocabularies into an application language by interpreting (at least some of) the elements of the language as being surrogates of the terms of the source vocabularies. 

This deﬁnition formalizes, with respect to Metathesaurus frames and frame sets, the notion of a term repre­sentation as described in section 3.4 and ensures that under that representation, the application language “uses” the terms in the vocabularies. It is possible (by clause (1)) that there are terms in the set of vocab­ularies that do not have surrogates in the application language – meaning that a set of thesauri (or in fact any single thesaurus) may contain more terms than are used in a given application language. Likewise, a given language may contain terms which “come from” multiple thesauri (frames), as in a case where drugs, medical condition, laboratory tests, and clinical procedures are all referred to in the application language. 

### 4.2 Representing concepts 

The time has come to consider how concepts are represented in the Metathesaurus, and consequently how they should be represented in our formalization of it. As we have seen in Section 2, even though the preponderance of Metathesaurus literature insists that synonymy is a relation among terms, it is not fundamentally so – instead being a fundamental relation among occurrences of terms, instances of terms, or (in the Metathesaurus) atoms. Likewise, then, when it is said in this literature that a concept is a “set of synonyms” or a “synonym class”, we now understand that this must mean that a concept is to be thought of as a set of *atoms* related by synonymy (as this is explicit in Fung, et al. (2005)). 

Such an approach to representing a concept as a synonym class has been suggested previously by Hilary Putnam, but he is careful to say that this is not intended to suggest that a concept is a synonym class. Rather, the suggestion is that for *certain purposes* it is convenient and fruitful to *think of a concept as a class of synonyms*:

I do not maintain that concepts are synonymy-classes, whatever that might mean, but that they can be identiﬁed with synonymy-classes, for the purpose of formalization of the relevant discourse. (Putnam (1967))

This is as well how we should interpret claims in the literature of the Metathesaurus to the effect that concepts are sets of synonyms (or sets of synonymous atoms). Even in doing so, however, we should replace this relatively simplistic view of a concept as a set of atoms with a more sophisticated representation under which the simpler one falls. 

There is a lengthy history in formal semantics of viewing a concept (formally) not as a set, but as a function or rule that “picks out” such a set in each appropriate context. Thus, for example, the concept of the King of England is thought of as a rule (or function) that picks out the individual who at each particular time is King of England at that time (and picks out no one if there is no King of England at that time). Thus under this construal the concept of being King of England is not just the set of all people who happen to have been the King of England, but is a more abstract thing (rule, set of criteria, etc.) that allows the identiﬁcation of the King of England in any particular circumstance. Similarly, the concept expressed by the term ‘Tetralogy of Fallot’ may be thought of as a rule that, in any possible world or set of circumstances, picks out all those cases which are cases of a particular congenital cardiac defect associated with four anatomical abnormalities. We can trace the origin and evolution of this formal representation of concepts at least back to Frege (1891, 1892), and its further development through Carnap (1947), Kaplan (1968), Montague (1970a), Scott (1970), et al. I will not recount here the century of analysis, application, criticism, and dispute involving this approach, but will urge that in the context of concepts and their uses in the Metathesaurus, there is much to recommend it for a couple of reasons. 

First, as we have seen in our discussion of how synonymy is determined in integrating a source into the Metathesaurus, it is clear that the determination of the synonymy relation – as well as decisions concerning when new atoms or concepts must be introduced, or when existing concepts should be merged – is based on the fundamental view that there is a community of competent language users who share a set of medical concepts and a use of medical terms to express these concepts. And at times the Metathesaurus literature (e.g., Fung, et al. (2005)) makes speciﬁc reference to rules or guidelines to be used in making the necessary assessments of synonymy or atomicity. In turn, this indicates that an adequate account of concepts in the Metathesaurus requires us to view concepts not merely as sets, but as rules or functions in the sense of Frege, Carnap, et al. 

Second, if a concept is taken to be just a set (of terms, synonyms, atoms, etc.), then whenever that set is changed for any reason, the concept has changed as well. This leads to the unintuitive consequence that whenever we discover something new to which a well-established concept clearly applies, we do not so much recognize that the new entity falls under our well-established concept as that we must view ourselves as changing (expanding) the concept to accommodate the new entity. And this in turn means that we cannot regard concepts as having any sort of transcendence over the terms associated with them – which is quite incompatible with the view and intended use of concepts in the Metathesaurus. In the Metathesaurus, concepts are regarded as abstract entities that are language-independent and that “tie together” terms in various languages/sources in virtue of the view that the concepts represent the meanings of the terms in those languages. The Concept Unique Identiﬁer (CUI) that represents a concept in the Metathesaurus is not changed whenever a new term (from either a new release of an existing source or from a new source being integrated) is added to that concept. The concept (CUI) remains in place – in a signiﬁcant sense, remains unchanged. It is only its set of associated terms (or atoms) that have changed in such an event; and this shows that the concept must be considered to be something different from simply a set of such terms (or atoms). It is precisely analogous to the intension (meaning) of a term remaining constant while its extension (denotation) changes from one context, index, or possible world to another. 

It is worth noting at this point that although discussions of concepts in the Metathesaurus literature frequently take such an intensional view of them, their implementation in the Metathesaurus data models is purely extensional. It is in the data model that a concept appears to be only a set of atoms associated with a Concept Unique Identiﬁer (CUI), rather than a rule or function. It need not be so. For example, rather than representing the Metathesaurus as simply a set of records in a relational data base, and representing each concept in terms of a CUI that appears in such (purely extensional) records, it would be possible to represent a concept (“named” by its CUI) as a function or procedure that, on given input, would generate its “extension” (the set of records in which it occurs and which relate it to the atoms it represents). Such an approach would explicitly represent a Metathesaurus concept as a rule (intension). In fact, there is– in discussions of the automated alignment of sources – evidence to suggest that this is the underlying representation of concepts for the Metathesaurus, though it appears only in the code that the NLM uses to generate the sets of atoms for Metathesaurus sources and not in the purely extensional data base representation that is distributed as a Metathesaurus release.^18^

The view I am proposing here of seeing Metathesaurus concepts truly as rules or functions (following Carnap, et al.) is at odds with the interpretation imposed in Smith (2006) which he takes to be based on what is said in Campbell, et al. (1998). However, I think that Smith has been misled in this regard by the remarks he cites. Campbell, et al. (1998) in fact conﬂates the data representation or data model of the Metathesaurus with the proper semantic representation of concepts in the Metathesaurus. But as I have just argued, while the data model is purely extensional, the semantics of concepts is intensional – though this is concealed from Metathesaurus users since it appears in a combination of the program code that implements automated alignment and guidelines for determining “true synonymy”. When Campbell et al. say “We argue that the ‘meaning’ of this identiﬁer is only understandable extensionally, ...”, this is a highly misleading assertion; but nowhere do they equate meaning with extension, and most of Campbell, et al. (1998) is devoted to an intensional view of meaning (though I cannot argue in support of the overall coherence of the view that emerges). 

Thus while for some purposes related to the Metathesaurus it may be convenient to adopt a view that concepts can be thought of simply as sets of atoms, such a perspective must be taken in Putnam’s sense as a pragmatic association and not to imply that concepts really are (or should be thought of as being fully modeled by) nothing more than synonym sets. In fact, such a simplistic direct association of a concept with a set of atoms or terms is a convenience (perhaps a necessary one, given certain constraints) for the purposes of using the Metathesaurus in computational contexts and for computational (e.g., information retrieval, classiﬁcatory, or data mining) purposes. 

### 4.3 Truth, synonymy, and concepts: a formal approach 

Metathesaurus source frames and application languages are missing some critical elements and detail. While they provide at least some formal representation of source vocabularies, strings, atoms, terms, and relationships as these appear in Table 1, they provide no insight into concepts or synonymy. To remedy these ﬂaws, we will introduce the notion of a Metathesaurus *model*; but ﬁrst we must devote some attention to truth. 

While UMLS 2 and UMLS 5 characterize synonymy through an appeal to the interchangeability of terms in sentences, this approach raises an immediate problem in the context of the Metathesaurus because of the existence of ambiguous terms: depending upon which sense of the ambiguous term is considered, the very same sentence may be true on one reading and false on another. And such a situation is more than a mere logical possibility in the case of the Metathesaurus. A simple example is to be found with the term ‘mole’, which may refer to either (a) a malformation of the skin, (b) a malformation of the skin speciﬁcally containing melanin, (c) a small burrowing animal, (d) a unit of measurement, or (e) the DUOXA1 gene. 

What, then, is the truth value of the sentence “A mole is an insectivore.”? Well, it is true on reading (c), but false on (a), (b), (d), and (e). Moreover, the indeterminacy of the truth of this sentence is not helped in the Metathesaurus even if we conﬁne ourselves to a single source vocabulary (or, in our representation, a single Metathesaurus frame) since, for example, the SNOMED-CT source itself includes senses (a), (b), and (c) of the term ‘mole’ (making ‘mole’ ambiguous within SNOMED-CT). Thus relativizing the truth of sentences to (informally) sources or (formally) frames would not in itself eliminate the ambiguity of sentences and assure that we could assign meaningful truth values to them. And failing to distinguish the senses in which a sentence can be true may then further lead to faulty inferences such as

Some moles develop into melanoma and should be removed.
Moles may be removed by the application of Mole Med (a product for treating lawns and eliminating moles).
Therefore, this mole on my skin should be treated with Mole Med.

Many other less humorous and potentially more dangerous inferences can be made if care is not taken to distinguish the different senses in which a term may be used.

There are several possible approaches to addressing the truth values of sentences in our application languages, and the one we shall take here has certain advantages in simplicity and in allowing us to con­strue the logic associated with these languages to be classical sentential or ﬁrst-order predicate logic. In pursuit of those goals we will constrain the notion of truth to apply, ﬁrst, to sentences only in our application languages; second, to be relativized to frames; and third, to apply only to disambiguated sentences. 

A truth value – at least in a standard bivalent semantics, which we assume here – cannot be assigned to a sentence which is ambiguous, since on one meaning the sentence might be true while on another it might be false. And this approach to avoiding ambiguity is also consistent with the requirement of NISO (2005), section 5.3.1, that terms in a thesaurus are not permitted to be ambiguous. We must consequently have some mechanism of disambiguating sentences that contain ambiguous terms, and for this purpose we shall introduce the notion of a disambiguating function. But doing so requires ﬁrst that we turn our attention to a formal representation of concepts. 

In light of our previous discussion of these as they appear in Metathesaurus literature, we take a concept to be represented formally in the manner of Frege, Carnap, et al. as a function that picks out in each situation the set of things that realize or instantiate the concept in that situation. In a standard referential model-theoretic semantics, this would make our notion of concept language-independent. In our current context, it can still be seen as language-independent in a sense – which is that it is not directly deﬁned with respect to a term that represents the concept. 

**Deﬁnition 6**. If 𝔉^∗^ is a Metathesaurus frame set, then 𝔠 is a concept in 𝔉^∗^ if and only if 

(1) 𝔠 is a function whose domain is 𝔉^∗^, and 

  (2) for each 𝔉  in 𝔉^∗^ , 𝔠(𝔉 ) ⊆ O_𝔉_ . 

*Informally*: A concept is a rule that “picks out” in each frame the atoms of the frame to which the pconcept applies, or picks out the empty set if the concept applies to no atom in the frame . 19

**Deﬁnition 7**. If 𝔉^∗^ is a Metathesaurus frame set, then 

(1) 	Concepts_𝔉^∗^_ = {𝔠:𝔠 is a concept in 𝔉^∗^}. 

*Informally*: The concepts of (the frame set) 𝔉^∗^ .         

(2) If 𝔉  ε 𝔉^∗^ , α is an atom of 𝔉 , and 𝔠 is a concept in 𝔉^∗^, then 

(a) α realizes 𝔠 in 𝔉  if and only if α ε 𝔠(𝔉 ), and 

  (b) α is unrealized in 𝔉  otherwise. 

Note that this deﬁnition of ‘concept’ does not deﬁne or represent only the “reasonable” or “natural” concepts of a frame set, but rather what might be thought of as the “possible” concepts – which would include what Descartes might call “ﬁctional” concepts. It is a purely formal representation of concepts as functions or "rules". Under this construal, a concept is not necessarily associated with a term (no term related to the concept is mentioned in the deﬁnition) and may in fact lack any name. That is, a concept is conceived of as an abstract relation (function or rule) rather than a name in an application language; and an additional consequence of this is that concepts are not dependent upon any relation of synonymy for their formal characterization. (In the Metathesaurus, of course, each concept of interest has a canonical name – its Concept Unique Identiﬁer – but this leaves open the question as to whether there are unnamed concepts or undiscovered concepts which at a later time may be named and thus appear in a new release of the Metathesaurus.) At this point we must acknowledge that we are deviating from one fairly common view (found in the history of philosophy and logic) that a concept is associated with a term in that it can be thought of as the meaning or sense of the term. Such a view is reasonable provided that terms are not permitted to be ambiguous; but given that term ambiguity is a rather essential feature of the Metathesaurus, we cannot think of a concept as representing the meaning of a term. Instead, in the context of the Metathesaurus, a concept is to be thought of as representing the meaning of an atom (or of a certain set of atoms), and may be thought of as representing a (one possible) meaning of a term. 

This leads us to the notion of a concept assignment for a frame set: 

**Deﬁnition 8**. If 𝔉^∗^ is a Metathesaurus frame set, then C is a concept assignment for atoms in 𝔉^∗^ if and only if 

(1) C is a function whose domain is Atoms(𝔉^∗^), and 

  (2) for each α ε Atoms(𝔉^∗^) 

 (a) C(α) is a concept in 𝔉^∗^ and 

 (b) for some 𝔉  ε 𝔉^∗^ , α realizes C(α) in 𝔉 . 

*Informally*: A concept assignment associates a single concept with each atom – specifying one interpretation of that atom’s term in such a way that the atom realizes that concept. 

If C is a concept assignment for atoms in 𝔉^∗^, then Clause (2)(a) and Deﬁnitions 3, 6, and 7 ensure that C(α)(𝔉 ) is a set of atoms in 𝔉 . And Clause(2)(b) ensures that an atom is always one of the things assigned by its concept to the frame in which it occurs (recalling that each atom occurs in only a single frame). Less formally, this means that when a concept is associated with an atom, that atom is “in” the concept – that is, that the atom is one of the atoms realized by the concept. And in turn, this ensures that an atom will be synonymous with itself. If concepts are thought of as meanings, then a concept assignment for atoms may be thought of as a rule that associates a single speciﬁc meaning with each atom in the Metathesaurus. It therefore interprets the atoms and indirectly associates terms with concepts (since each atom is itself associated with a unique term) – but with the caveat that while an atom is associated with only one concept, a term may be (ambiguously) associated with multiple concepts through its different atoms. 

### 4.4 Metathesaurus models 

We may now begin to tie together our characterizations of terms, atoms, concepts, and synonymy by introducing Metathesaurus models. This will be done in several stages – partly for pedagogical reasons, and partly to emphasize the role and nature of synonymy in the Metathesaurus as well as the relation of the Metathesaurus to potential applications. 

#### 4.4.1 Minimal Metathesaurus Models 

A minimal Metathesaurus model is simply a frame set together with a concept assignment for atoms. 

**Deﬁnition 9**. 𝔐= (𝔉^∗^ , C) is a minimal Metathesaurus model if and only if 

(1) 𝔉^∗^ is a Metathesaurus frame set, and 

(2) C is a concept assignment for atoms in 𝔉^∗^ . 

Minimal Metathesaurus models provide us with a formal characterization of source vocabularies and of relations among terms, atoms, and concepts. They are minimal in the sense that they impose virtually no constraints on concept assignments and hence on what concepts there are from the perspective of the model. It is quite possible, for example, that in a minimal model of this sort each of the terms ‘Heart failure’, ‘Tooth decay’, and ‘Pregnancy’ would be associated with the same concept. Such a concept would correspond to that of a ﬁctitious idea, in the sense of Descartes, in that it lacks “objective reality”. However, even on this minimal basis we can deﬁne some additional important notions. 

**Deﬁnition 10**. If 𝔐= (𝔉^∗^ , C) is a minimal Metathesaurus model, then Concepts_𝔐_ = Range(C). 

*Informally*: The concepts of a model are all the concepts associated with any of its atoms (by its concept assignment). 

**Theorem 1**. If 𝔐= (𝔉^∗^ , C) is a minimal Metathesaurus model, then Concepts_𝔐_ ⊆ Concepts_>𝔉^∗^_. 

*Informally*: The concepts of a model are among the set of possible concepts for its frame set. 

**Deﬁnition 11**. If 𝔐= (𝔉^∗^ , C) is a minimal Metathesaurus model, then 

(1) α realizes c in 𝔐 if and only if α realizes 𝔠 in 𝔉  for some 𝔉  ε 𝔉^∗^.   

Informally: An atom realizes a concept – under a given concept assignment – if it is one of the items “picked out” by the concept in some frame. 

(2) If τ ε Terms(𝔉^∗^), then τ expresses 𝔠 in 𝔐 if and only if there is some atom α ε Atoms(𝔉^∗^) 

(1) α realizes 𝔠 in 𝔐, and 

(2) α exempliﬁes τ in 𝔉^∗^ . 

Informally: A term expresses any concept that is realized by (or “picks out”) any of its atoms. 

**Figure 1:**
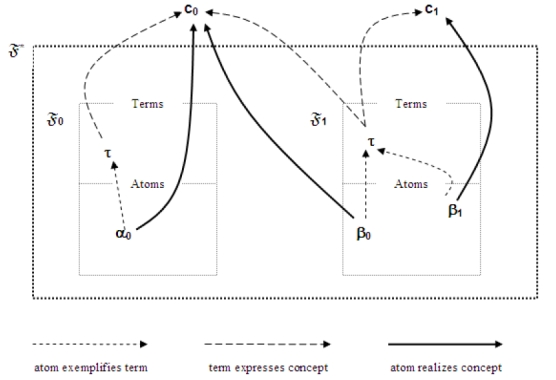
Basic relations among terms, atoms, and concepts in models

Figure 1 is offered as an aid to the intuitions. It illustrates a frame set 𝔉^∗^ with two frames, 𝔉 _0_ and 𝔉 1. A term τ occurs in both frames, with a single occurrence (atom) α_0_  in 𝔉 _0_  and two occurrences (β_0_  and β1) in 𝔉 _1_ . The term τ is exempliﬁed by α_0_ , β_0_ , and β_1_. β_1_ realizes the concept c_1_ while both α_0_  and β_0_  realize the concept c_0_ . A concrete example of such a situation is found when 𝔉 _0_  is taken to be MedDRA, 𝔉 _1_ is taken to be SNOMED-CT, and τ is taken to be ‘cold’. MedDRA recognizes ‘cold’ to have a single meaning while SNOMED-CT countenances multiple meanings, one of which is shared with MedDRA. 

The following theorem demonstrates that minimal Metathesaurus models impose the correct properties on the relations of exempliﬁcation and realization (in accord with NLM (2008)):

Theorem 2. If 𝔐= (𝔉^∗^ , C) is a minimal Metathesaurus model, then 

 (1) if α ε Atoms(𝔉^∗^), then there is a unique τ ε Terms(𝔉^∗^) such that α exempliﬁes τ in 𝔐. Informally: An atom exempliﬁes exactly one term. 

  (2) if τ ε Terms(𝔉^∗^), then there is some α ε Atoms(𝔉^∗^) such that α exempliﬁes τ in 𝔐. Informally: Every term is exempliﬁed by at least one atom. 

  (3) if α ε Atoms(𝔉^∗^), then there is a unique 𝔠 ε Concepts_𝔐_ such that α realizes 𝔠 in 𝔐. Informally: An atom realizes exactly one concept. 

#### 4.4.2 Ambiguity and a surfeit of synonymies 

We now have, in the context of minimal Metathesaurus models, several fairly intuitive formal representations of relations among terms, atoms, and concepts. First, an atom exempliﬁes a term: it is one instance (exemplar) of its term as this is associated with a particular meaning and use. And multiple atoms may exemplify the same term. 

But an atom realizes a concept: it is one of the things that falls under or is comprehended by the concept. And a concept may (typically does) comprehend a number of such realizations which are referred to by means of distinct terms. For example, the concept of heart failure may comprehend a set of atoms (in the ICD-9 CM, MedDRA, and Metathesaurus ICD-9 source vocabularies) whose terms include ‘Heart failures’, ‘Heart failure’, ‘Heart failure NOS’, ‘Heart failure, unspeciﬁed’, ‘Weak heart’, ‘Cardiac insufﬁciency’, ‘Heart insufﬁciency’, ‘Cardiac function failed’, ‘Insufﬁciency -cardiac’, etc. 

Finally, a term expresses a concept if at least one of its atoms realizes that concept. Thus in the Metathe­saurus the term ‘Heart failure’ expresses the concept whose CUI is C0018801 since its atom A8339687 (in the ICD-9 CM source vocabulary) realizes that concept. Other atoms of ‘Heart failure’ also realize C0018801. These include A0001705 in MedDRA and A4708533, A4708534, A2872568, and others in the SNOMED-CT source. 

Because a term may be exempliﬁed by multiple distinct atoms having different senses (i.e., may be associated with different CUIs), a term may express more than one concept. This leads us to one deﬁnition of the property of ambiguity in our models as: 

Deﬁnition 12. If 𝔐= (𝔉^∗^,C) is a minimal Metathesaurus model, then τ is unambiguous in 𝔐 if and only if all atoms that exemplify τ in 𝔉^∗^ realize the same concept in Concepts_𝔐_. And τ is ambiguous in 𝔐 if and only if it is not unambiguous in 𝔐. 

*Informally:*A term is ambiguous if it expresses multiple concepts. Otherwise it is unambiguous. 

Thus in Fig. 1 τ is ambiguous since it expresses both 𝔠_0_ and 𝔠_1_. 

We may even, at this point, and based on our notion of minimal Metathesaurus model, introduce formal characterizations of some notions of synonymy. The synonymy relation for atoms in terms of concepts is straightforward because atoms cannot be ambiguous: 

**Deﬁnition 13**. If 𝔐= (𝔉^∗^, C) is a minimal Metathesaurus model, α εAtoms(𝔉^∗^), and β εAtoms(𝔉^∗^), then α is atom-synonymous with β in 𝔐 if and only if there is a 𝔠 εConcepts_𝔐_ and both α and β realize 𝔠 in 𝔐. 

*Informally *(And since by Theorem 2(3), every atom realizes exactly one concept.): Two atoms are atom-synonymous just in case they realize the same concept. 

So, in Fig. 1, α_0_ and β_0_ are atom-synonymous because both realize the concept 𝔠_0_. And atom-synonymy is an equivalence relation: 

**Theorem 3**. If 𝔐= (𝔉^∗^ ,C) is a minimal Metathesaurus model, α ε Atoms(𝔉^∗^), β ε Atoms(𝔉^∗^), and γ ε Atoms(𝔉^∗^) then 

(1) (Reﬂexivity) α is atom-synonymous with α.  

(2) (Symmetry) If α is atom-synonymous with β, then β is atom-synonymous with α.  

(3) (Transitivity) If α is atom-synonymous with β and β is atom-synonymous with γ, then α is atom-synonymous with γ.  

Clause (1), reﬂexivity, depends on Clause (2) of Deﬁnition 8.  Otherwise, it and the clauses for symmetry and transitivity follow quite directly from previous deﬁnitions. 

However, since terms may be ambiguous, characterizing a synonymy relation among them in minimal Metathesaurus models is more challenging and the results are less satisfying. As a more thorough inspec­tion of our models reveals, this is primarily because there is a multiplicity of relations among terms that all have some claim to being called “synonymy”, and there are no clear grounds in all circumstances for selecting one of these to be the synonymy relation: 

**Deﬁnition 14**. If 𝔐= (𝔉^∗^ , C) is a minimal Metathesaurus model, τ ε Terms(𝔉^∗^), and η ε Terms(𝔉^∗^), then 

(1) τ is partially term-synonymous with η in 𝔐 if and only if some exemplar of τ is atom-synonymous with some exemplar of η in 𝔐. 

Informally: τ and η are partially term-synonymous if and only if they “overlap” in meaning. 

(2) τ is strongly term-synonymous with η in 𝔐 if and only if τ and η express exactly the same concepts in 𝔐. 

   *Informally *: τ and η are strongly term-synonymous if and only if every meaning of τ is also a meaning of η; and every meaning of η is a meaning of τ. 

(3) τ and η are unambiguously term-synonymous if both are unambiguous and express the same concept in 𝔐.

*Informally *: τ and η are unambiguously term-synonymous if and only if they are each unambiguous and have the same meaning. 

**Figure 2:**
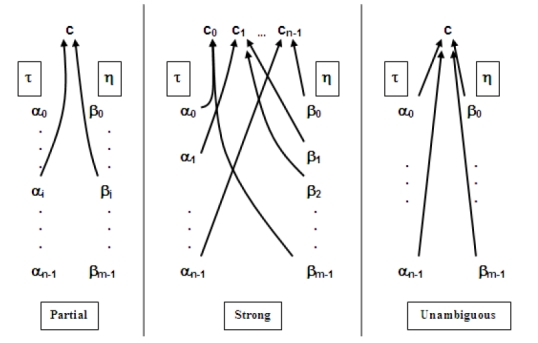
Three possible synonymy relations among terms

Partial term-synonymy and strong term-synonymy are similar, respectively, to the notions of weak synonymy and strong synonymy described in Montague (1970b). Additionally, strong term-synonymy illustrates the type of synonymy among terms discussed in Section 2.2. Fig. 2 provides visual illustrations of these different synonymy relations where τ and η are terms, the cis are concepts, the αis and βis are atoms (of τ and η respectively), and arrows represent the relation of realization between atoms and concepts. Both strong and unambiguous term-synonymy are, like atom-synonymy, equivalence relations. But partial term-synonymy is not, failing to be transitive. Alas, there are even more synonymy relations among terms than Deﬁnition 14 and Figure 2 suggest since each of those gives rise in turn to a variety of restricted synonymy relations where the relation holds only for a subset of frames in a given frame set. 

Partial term-synonymy will not guarantee the truth-preserving interchange of synonyms in all contexts; and the likelihood that two terms will be strongly or unambiguously term-synonymous while differing in anything other than a trivial lexical manner is remote indeed. Unambiguous term-synonymy will ensure in­terchange of synonyms salva veritate. Strong term-synonymy will as well, but in an odd way since the result of such a substitution may well be (or remain) ambiguous. Even if, in a given release of the Metathesaurus, two terms are as a matter of fact strongly term-synonymous or unambiguously term-synonymous, there is no guarantee that a later source added to the Metathesaurus will not use one of those terms in a different sense – breaking the synonymy relation. 

Minimal Metathesaurus models succeed in modeling relationships among terms, atoms, and concepts in the Metathesaurus – particularly as these appear in the data base tables that comprise a release of the Metathesaurus. But although they allow us to deﬁne certain relations of synonymy by means of concepts, they do not suggest how these notions are related to the scientiﬁc world or to an objective and scientiﬁcally meaningful synonymy relation. For this, we need something a bit more complex. 

#### 4.4.3 Synonymy Models 

One of the fundamental problems with minimal Metathesaurus models is that they in no way address the problem of the ambiguity of terms, so that it is far from clear how the notion of truth could coherently be deﬁned relative to such models. In addition, they provide no basis for an account of how – in practice – the concept assignment for a model (or the set of concepts countenanced by a model) may be determined. As a step in this direction, we must ﬁrst provide a formal model of what we may think of as the empirically determined synonymy relations that form a central part of the use of biomedical vocabularies and are referred to in such treatments as McCray and Nelson (1995) and Fung, et al. (2005) as justifying the concept structure of the Metathesaurus. To do this, we will assume that the notions of a term,a thesaurus as a set of terms, an application language,a term representation, and a truth-value interpretation may be explicated and formally deﬁned along the lines suggested in Section 3.4. Then 

**Deﬁnition 15**. 𝔖= (F^∗^ , 𝔏, r, t, S) is a term-based synonymy relation model if and only if 

(1) 𝔉^∗^ is a Metathesaurus frame set, 

(2) 𝔏 is an application language, 

(3) r is a term representation for Terms(𝔉^∗^) in 𝔏

(4) S is a binary relation in Terms(𝔉^∗^), and 

(5) For any τ and η in Terms(𝔉^∗^), 

   if (τ, η)εS and ϕ is a sentence of 𝔏,

  then 

  t(ϕ)= t(ϕ^i^_ r,τ,η_) for any i. 

  Informally: The Interchangeability of Synonyms holds in the application language when term-surrogates are substituted for synonymous thesaurus terms. 

Note that for this deﬁnition we do not need to employ the notion of a Metathesaurus frame set, but could as easily make use of the weaker notion of a thesaurus as a set of terms (since it is only the terms in the frame set that play a role in the deﬁnition). However, there is no loss of generality in appealing to frames and a frame set in this case, we know from previous discussion that frame sets represent sets of thesauri in the relevant ways, and making use of frame sets in this context will facilitate subsequent deﬁnitions and discussion. 

This quite simplistic characterization of a synonymy relation ensures only that synonymy, so construed, is a relation among terms and that synonyms are interchangeable salva veritate in the sentences of a language (i.e., that the principle of the Interchangeability of Synonyms is satisﬁed). However, it thus serves satisfactorily as a formal model of the synonymy relations appealed to in Metathesaurus literature that are employed to generate or to justify the set of Metathesaurus concepts as meanings of terms. And it will allow us to characterize with further precision the relation of synonymy to terms, atoms, and concepts in the Metathesaurus. 

#### 4.4.4 Synonymy-based Metathesaurus models 

We can now introduce synonymy-based Metathesaurus models and then demonstrate how such models provide insights and solutions to the problems and questions with which this paper began. But ﬁrst we require the notion of a disambiguating function. 

**Deﬁnition 16**. If 𝔐= (𝔉^∗^ , C) is a minimal Metathesaurus model, then d is a disambiguating function (or disambiguator) for 𝔐 if and only if 

(1) d is a function whose domain is Terms(𝔉^∗^), and 

(2) for each τ ε Terms(𝔉^∗^), d(τ) ε Concepts_𝔐_. 

*Informally*: A disambiguating function assigns to each term a unique concept. 

The intention here is that a disambiguator interprets a (possibly otherwise ambiguous) term in one unambiguous sense across all of the frames in a frame set. 

**Deﬁnition 17**. 𝔐= (𝔉^∗^ , C, r, t, S, d) is a synonymy-based Metathesaurus model if and only if 

(1) (𝔉^∗^ , C) is a minimal Metathesaurus model, 

(2) d is a disambiguating function for (𝔉^∗^ ,C), 

(3) (𝔉^∗^ , 𝔏, r, t, S) is a term-based synonymy relation model, and 

(4) for any terms τ and η, (τ, η) ε S if and only if d(τ)= d(η). 

Informally: Two terms are synonymous if and only if they (unambiguously) express the same concept. 

Note that a synonymy-based model cannot be deﬁned without reference to a synonymy relation, which in turn cannot be deﬁned without reference to an application language and a truth-value interpretation of that language. But what, exactly, do we take the term-based synonymy relation S to represent? We take this to represent a source-transcendant relation of synonymy among terms that results from a consensus of biomedical language users together with informed judgements made by Metathesaurus curators as to whether two terms should be regarded as synonymous in this transcendant sense. Thus synonymy-based Metathesaurus models expose the essential relation between the concept of (transcendant) synonymy as it appears in Metathesaurus literature and the application of the Metathesaurus by means of an application language. In addition, the notion of a synonymy-based model requires an appeal to a disambiguating function; and this is a formal reﬂection of the realization discussed in Sections 2.1-2.3 that strict synonymy among terms makes sense only when those terms are not ambiguous. Finally, this deﬁnition reﬂects our decision in Section 2.2 to regard synonymy as a primitive semantic relation. 

## 5 Beneﬁts of this approach 

Looking at Table 1 in Section 3, we can see that our formal models accommodate the basic elements of the Metathesaurus. They also provide accounts of ambiguity and synonymy, and of application languages in their relation to thesauri. Beyond that, Sections 1.1 and 1.3 introduced some puzzles and questions concerning the relationships of terms, atoms, concepts, and synonyms in the Metathesaurus. The formal analysis developed in Section 4 should now serve us in in addressing those puzzles and questions with a high degree of precision. 

### 5.1 Terms, concepts, and synonyms: the fundamental relations 

The answer to Question 1 (“What is a term? What, in the context of the Metathesaurus and explications of it, does ‘term’ mean?”) is quite complex – more so than we had expected at the outset. Indeed, the term ‘term’ in the Metathesaurus – and more generally in medical informatics – is itself highly ambiguous and subject to severe equivocation even within the same publication, sometimes meaning ‘string’ (sequence of characters), sometimes ‘lexical equivalence class of strings’, and sometimes ‘string of a lexical equivalence class with certain semantic or pragmatic attributes’. Keeping these different senses of ‘term’ both clear and distinct can be a constant struggle in reading the literature pertaining to the Metathesaurus and in infor­mal discussions about it. However, the Metathesaurus documentation itself draws the technical distinctions clearly on the basis of what SUIs (String Unique Identiﬁers), LUIs (Lexical Unique Identiﬁers), and AUIs (Atom Unique Identiﬁers) represent. Such distinctions become critical in the context of any account of syn­onymy or any treatment of ambiguous terms – for as we have seen, it is not really terms that can reasonably be said to be synonymous, but rather occurrences (or contextual interpretations) of terms, represented in the Metathesaurus as atoms. 

This leads also to an answer to Question 2 ("What is the role of term types in characterizing and indi­viduating terms?"). While we have made no attempt at a detailed analysis of the nature of atoms in the Metathesaurus, we have indicated that atoms are distinguished (individuated) through a variety of attributes that terms (i.e., strings or lexical equivalence classes of strings) may have. Some of these attributes are lexical (and include the lexical type of the term such as noun or adjective), some are semantic, and some are pragmatic (which may include a type indicator for how the term is used in a given vocabulary – such as the distinction in MedDRA between a Lowest Level Term and a Preferred Term having the same string representation). 

Question 3 ("What is the role of atoms in characterizing terms, concepts, and synonymy?") and Question 4 ("Can a term be ambiguous? What effect does this have on how concepts and synonymy can be charac­terized?") turn out to have highly complex answers, the formal renditions of which appear in our explication of the relations of exempliﬁcation (between an atom and its term), realization (between an atom and its concept), and expression (between a term and a concept). We can see that Section 4 provides a detailed account of precisely what it means to say that a term expresses a concept while its atoms exemplify the term and realize the concept – while avoiding the difﬁculties introduced by ambiguity. This also provides us with precise answers that are raised in questions that arise in reading the Metathesaurus literature:

How, in the Metathesaurus, does a term “express the meaning of the concept” (UMLS 9)?

Answer: A term expresses the meaning of a concept when one of its (unambiguous) atoms realizes the concept. In minimal Metathesaurus models this is represented by the concept assignment to atoms C, and in synonymy-based Metathesaurus models it is represented by means of C together with the disambiguating function d. Since a term may have multiple atoms, some of which have one sense and some of which have another, a term may be ambiguous in that (without disambiguation) it expresses the meaning of multiple concepts.

What does it mean to say that “synonymous names are joined in a concept” (UMLS 1), and in precisely what way is it that “synonymous terms from various terminologies are clustered into concepts” (UMLS 6)?

Answer: In the context of synonymy-based Metathesaurus models, this is given precise meaning in the sense that all terms that are deemed to be synonymous (i.e., that enter the synonymy relation S with one another) are assigned, as their meaning, the same concept. It is in this way that the Metathesaurus represents – by means of its concepts and their relation to atoms – the use of terms by the biomedical community.

Answer: In the context of synonymy-based Metathesaurus models, this is given precise meaning in the sense that all terms that are deemed to be synonymous (i.e., that enter the synonymy relation S with one another) are assigned, as their meaning, the same concept. It is in this way that the Metathesaurus represents – by means of its concepts and their relation to atoms – the use of terms by the biomedical community.

In what way do synonyms “carry the same meaning” (UMLS 1) and how is it that “each of the terms in a synonym class represents exactly the same meaning" (UMLS 2)?

Answer: Synonymous atoms carry the same meaning in virtue of the fact that they realize the same concept – which is the meaning they carry. This relationship is expressed formally in Deﬁnition 13. We have identiﬁed three cases (Deﬁnition 14 and Fig. 2) in which terms may sensibly be said to be synonymous. In the ﬁrst case, two terms may be partially synonymous if an atom of the one term is atom-synonymous with an atom of the other. This means, roughly, that one use of the ﬁrst term is synonymous with some use of the second – though other uses may not be synonymous. Or they may be strongly synonymous in the unlikely event that each of their atoms is atom-synonymous with an atom of the other term – which means that every use of the ﬁrst term reﬂects a corresponding use of the second term, and conversely. Or ﬁnally, they may be unambiguously synonymous if both are unambiguous and express the very same concept. In all of these cases, the synonyms “carry the same meaning” in virtue of the association of the same concept (or concepts in the case of strong term-synonymy) with their respective atoms.

Answer: A term may have several meanings if it is ambiguous: that is, if it has multiple atoms, each of which has a distinct and unambiguous meaning. In addition, while Deﬁnition 17 provides a representation of synonymy, terms, atoms, and concepts as related to only a single “external” synonymy relation, it is easy to see that it could be generalized to refer to a sequence S^∗^ of synonymy relations and a corresponding sequence d^∗^ of disambiguating functions. Such an extended synonymy-based model would then represent multiple disam­biguated synonymy relations among terms.

How may a term “have several meanings and belong to several concepts” (UMLS 8)?

Answer: A term may have several meanings if it is ambiguous: that is, if it has multiple atoms, each of which has a distinct and unambiguous meaning. In addition, while Deﬁnition 17 provides a representation of synonymy, terms, atoms, and concepts as related to only a single “external” synonymy relation, it is easy to see that it could be generalized to refer to a sequence S^∗^ of synonymy relations and a corresponding sequence d^∗^ of disambiguating functions. Such an extended synonymy-based model would then represent multiple disambiguated synonymy relations among terms.

What does it mean for ambiguous terms to be synonymous? Is it even possible for ambiguous terms to be synonymous? How can ambiguous terms appear in concepts since concepts are sets of synonyms?

Answer: These questions now ﬁnd precise answers in Deﬁnitions 12-14 and in the relations of terms to atoms explicated throughout Section 4. Ambiguous terms may be synonymous in at least two different senses: those of partial and strong term synonymy. They are related to concepts (as all other terms are) through their atoms. And these possibilities expose some of the pitfalls in inferencing in the Metathesaurus on the basis of “terms”. To go from a term in one vocabulary, through a concept expressed by that term, to a “synonymous” term in a second vocabulary requires substantial care in selecting the atom of the ﬁrst term that exempliﬁes it in the appropriate sense (i.e., realizes the appropriate concept). Without such care, faulty inferences can be made as a result of ambiguity or partial synonymy (recall from Section 4.3 the inference to treating skin lesions with rodent poison by incautious use of the mole concept).

In response to Question 5 (“In what way are UMLS concepts not universals (in the sense of Smith (2004))? Can they be made to serve the role of universals?”), we can see that there appears to be nothing in our explication of concepts that prohibits them from being universals – and that indeed, they seem to serve this function in the Metathesaurus. However, how well and completely they can serve in this role is another question that we will deal with below. 

### 5.2 Solving the puzzles 

The answers to the cluster of questions surrounding Question 3 also address the two puzzles illustrated in Section 1.1. Brieﬂy, these were

What is the difference between apparent synonymy and actual (or “true”) synonymy; and how is this related to precisely what it is (terms or other entities) that enter the synonymy relation?

and

If a concept forms an equivalence class under the synonymy relation, is a concept closed under the synonymy relation?

An important step in solving these puzzles is the realization that there is no single synonymy relation, but that instead there is a multiplicity of relations among atoms and terms that may be (and have been) called ‘synonymy’. Like ‘term’, in informal and imprecise contexts, ‘synonymy’ and ‘synonym’ are highly ambiguous (as we have demonstrated in the context of Deﬁnitions 13 and 14). Yet many users of the Metathesaurus continue to speak of “synonyms” and “synonymy” as though these have clear and unequivocal meanings. It is the failure to understand this that, more than anything else, inhibits a full understanding of the Metathe­saurus and an ability to make use of it in a reliable fashion. 

Consideration of our deﬁnition of synonymy-based models helps to provide an answer to the ﬁrst puzzle concerning a difference between a relation of “true synonymy” and one of only “apparent synonymy”. Each source vocabulary in the Metathesaurus may contain its own “local” or “parochial” view of the (or more precisely, a) synonymy relation. Such a relation may “work” when restricted to domain(s) of application originally envisioned or commonly encountered with respect to that source. Within such a constrained application environment, the Interchangeability of Synonyms may well hold. In fact, it ought to hold since the creators of the vocabulary in question surely have that in mind when deeming two terms to be synonyms. These local (or in Metathesaurus jargon, “source asserted”) synonymy relations are – from the perspective of the Metathesaurus – relations of apparent synonymy. They might also be regarded as relations of context-restricted synonymy in that terms are regarded as synonymous within a particular restricted context, but not necessarily as synonymous outside of that context. Such terms may not really be synonymous in the transcendant sense that they are synonymous in other vocabularies (or across vocabularies) as well. A simple example is found in SNOMED-CT which treats the terms ‘dog’ and ‘bitch’ as synonymous while the Metathesaurus assigns them to different concepts. (This and a variety of other examples may be found in Mills (1998).) Again, this is consistent with the picture of synonymy drawn in Section 2. 

We have chosen, for reasons of clarity and simplicity, not to represent such synonymy relations in our models. However, extending our models to such an explicit representation would require only the addition of a set S to each frame that would represent the source asserted synonymy relation for that frame. The Metathesaurus itself, in pursuit of its goal of “source transparency”, also represents such local synonymy relations – but not through its concepts (which represent only the transcendant relation). Rather, it represents them by means of a number of relation types (such as SY for “source asserted synonymy”) that appear in the representation of relations in its Related Concepts table (ﬁle MRREL.RRF) and are tersely and uninformatively described in the Relationship table of NLM (2008), Appendix B.3 (Abbreviations Used in Data Elements). 

Metathesaurus concepts are intended to represent the meanings of terms across multiple vocabularies or languages, and our minimal Metathesaurus models demonstrate how this is accomplished through the use of atoms and their relations to terms and concepts. We represent the source transcendant (or “true” or “real”) synonymy relation by means of disambiguating functions and term-based synonymy relations in synonymy-based Metathesaurus models; but that is enough for the purposes at hand. These models then provide us with a precise characterization of the relations of atoms, terms, and concepts that explicates how the Metathesaurus represents a source-transcendant sense of “synonym”. While this representation of synonymy by Metathesaurus concepts may be ﬂawed in various respects, synonymy-based Metathesaurus models provide an argument that it is coherent and may be used in applications if sufﬁcient attention is paid to the role of atoms as representatives of disambiguated terms. 

With respect to the question concerning whether concepts are closed under the synonymy relation, the answer to this is also now clear. If the synonymy relation is taken to hold among atoms, then the answer is “Yes.”, and is supported by the following theorem that holds in our minimal Metathesaurus models: 

**Theorem 4**. If α is atom-synonymous with β, then α realizes concept 𝔠 if and only if β realizes 𝔠. 

*Informally:* Any two synonymous atoms realize the same concept. Alternatively: concepts are closed under atom-synonymy. 

If the synonymy relation is taken to hold among terms, then there is no single answer since there is no single synonymy relation among terms, and the answer will in general depend on the precise synonymy relation being contemplated (e.g., partial, strong, or unambiguous) and whether it is being thought of in a restricted or unrestricted manner. However, there is one unequivocal relationship that can be proven on the basis of minimal Metathesaurus models: 

**Theorem 5**. If τ is unambiguously term-synonymous with η, then τ expresses concept 𝔠 if and only if η expresses 𝔠. 

Informally: Any unambiguous synonymous terms express the same concept. Alternatively: concepts are closed under synonymy for unambiguous terms. 

Note that the informal reading of this is a fairly loose interpretation of the theorem since concepts are in fact not made up of terms and hence cannot strictly be thought of as being closed under any kind of term synonymy. Signiﬁcantly weaker versions of this theorem can be proved for partial and strong term-synonymy, but they are of little interest. 

### 5.3 Concepts and reality 

Let us now turn our attention in more detail to arguments presented by Barry Smith in Smith (2004, 2006), since I believe that these can contribute more to our understanding of the nature of concepts in the Metathe­saurus and in addition will provide some insight into how our approach answers Question 6 (“Is the Metathe­saurus representation of terms, concepts, and synonymy truly divorced from empirical reality, or is there a clear relation between the Metathesaurus and the empirical world of biomedicine?”). Smith expresses sev­eral concerns about making use of concepts as fundamental elements in one’s ontology. He is concerned that “the intended role of concepts within ontology is itself subject to a variety of conﬂicting (and sometimes intrinsically incoherent) interpretations,” that it is “widely accepted that concepts are in some sense the products of human cognition,” and that too often “concepts become the very subject-matter of ontology”. Fundamentally, this is a concern that concepts have a highly psychologistic or subjective nature and hence are not appropriate entities on which to base an objective – not to mention scientiﬁc – characterization of the empirical world. The concern is genuine and reasonable; but it should not be a concern about how concepts appear and are used in the Metathesaurus. 

Smith allows that “Intuitively, a good ontology is one which corresponds to reality as it exists beyond our concepts.” But this is precisely what the Metathesaurus seeks to do – and succeeds in doing through its methodology of determining synonymy based on the shared use of language by the biomedical community. Certainly no account of concepts (or universals, or the use of terms) would be acceptable if it did not conform to such use by expert language users – for it is exactly that use of the language of biomedicine that also ﬁnds its expression in the empirically conﬁrmed and accepted theories of the biomedical domain. While it is true that “One is not capturing knowledge when one describes the beliefs widely distributed in certain cultures pertaining to concepts such as alien implant removal or Chios energy healing,” it is equally true that – if there is any knowledge to be captured at all – one is capturing knowledge when one describes the beliefs widely distributed in the biomedical community pertaining to how scientiﬁc terms are correctly used in reporting clinical trials, conducting experiments, and formulating theories. 

Thus in so far as the concepts of the Metathesaurus reﬂect this use of language by the biomedical com­munity, then they must correspond to reality (at least to the degree that current theories accurately reﬂect that reality) – and there is no higher criterion of success to be had in reﬂecting reality than conformance to (in Smith’s own words) “theories of objective reality developed by the natural sciences”. A similar argument seems to appear brieﬂy in Smith (2006). It would be at least mildly bizarre to argue – outside of a purely philosophical and metaphysical context – that an appeal to abstract entities such as universals provides a closer and more meaningful link to empirical reality than does an appeal to the accepted scientiﬁc use of biomedical language that is employed by working scientists directly to reference and characterize empirical phenomena in the biomedical domain. There deﬁnitely are ﬂaws in the treatment of concepts to be found in the Metathesaurus (and I will mention an additional one in the next section), but resting on a notion of concept that is psychologistic or subjective is not among them. And while Smith accuses the Metathesaurus Semantic Network of employing a circular deﬁnition of ‘concept’, this happens in a context (McCray (1993)) in which it is not a deﬁnition that is being proffered, but rather an informal explication or illustration. (I cannot in fact ﬁnd the precise example from the paper that Smith cites, but there is a similar one.) 

There is in addition a remarkable assertion to be found elsewhere in McCray (1993) that “Philosophers of language distinguish between the ‘intension’ and the ‘extension’ of a term. The intension is the manner in which a term is described, and the extension is what the term refers to in the real world.” Such a characterization is attributed to Lyons (1979). But this is, from the perspective of philosophy of language, a most odd account of the intension/extension distinction; and I know of no philosopher who would endorse it. It makes the intension of a term a description of the term – which is quite different from the characterization of intensions (following Frege, Carnap, et al.) that appears in Section 4. Lyons was in fact a linguist rather than a philosopher, and McCray’s suggestion is that of a linguist in characterizing an intension by means of the properties or attributes that are used in describing a term. This term-or language-oriented perspective is revealing in that it falls prey to Smith’s complaint that the focus is entirely on language without any link to reality. It is this term-oriented lexicographic ﬂavor that permeates discussions of the Metathesaurus and results in the sort of confusions we have earlier examined. 

Our formal treatment of Metathesaurus concepts in Section 4 is clearly non-circular and neutral with respect to the metaphysical nature of concepts, and in particular it exhibits no hints of psychologism, “ide­alism”, or subjectivity. At least in that regard it does not appear to fall under Smith’s criteria for “bad” ontologies. There is one more issue that Smith raises with respect to the sort of Tarskian or set-theoretic approach taken here, it is deserving of consideration, and we will examine it in the next section. 

## 6 Conclusions and open questions 

The approach to a formal semantics advanced in Section 4 is evidently only a sketch, but it is sufﬁcient to eliminate a number of confusions and to answer several signiﬁcant and puzzling questions concerning the relations among terms, atoms, concepts, and synonyms in the Metathesaurus – and to do so in a manner that is precise while not being bound to a particular data model or application implementation. It has also allowed us to deﬁne precise notions of synonymy among atoms along with several derivative notions of synonymy among terms, and to provide a clear explication of ambiguity. As a result we have been able to make sense of some confusing – and sometimes apparently contradictory – assertions in the Metathe­saurus literature, and to indicate the degree to which the Metathesaurus provides us with a coherent relation of synonymy that transcends individual source vocabularies. This formal approach has been particularly useful in drawing certain distinctions – such as between atom-synonymy and term-synonymy, and between different varieties of ambiguity – that are otherwise difﬁcult to characterize and to communicate. Likewise, the distinction between a thesaurus and its application language provides additional insight into the com­plexity involved in making use of a thesaurus for knowledge discovery or inference in the empirical sciences, at least in such areas as large observational data bases such as insurance claims, prescription histories, and electronic health records. 

Nonetheless, it must be recognized that this analysis has some signiﬁcant limitations. First, it is restricted only to what might be thought of as the basic roles of terms, atoms, and concepts in the Metathesaurus (primarily what is represented in the Concept Names and Sources table, or MRCONSO.RRF ﬁle). And I have largely ignored a host of relations represented in the Related Concepts table (MRREL.RRF), and completely ignored the hierarchical relations represented in the Computable Hierarchies (MRHIER.RRF). This last may be regarded as a particularly troubling omission since these relations provide a rich basis for inferencing while at the same time introducing one of the central confusions for UMLS users: the (incorrect) belief that Metathesaurus concepts themselves form a hierarchy. While I will have something to say on this topic very brieﬂy below, limitations of space have prohibited an extended and careful formal treatment of it here. Likewise, nothing has been said about semantic type information or about the “mapping” relations that are also represented in the Metathesaurus and which attempt to capture other relationships pertaining to similarity of meaning among terms. However, a full consideration of these topics would not alter the approach taken, or the conclusions reached here. 

Thus we may regard this as a preliminary study in providing a formal semantics for the Metathesaurus and as having provided a skeleton of a semantics designed primarily to aid in clarifying some of the funda­mental concepts on which the Metathesaurus is based. The discussion here can be viewed as an example of what can be done to achieve such goals, and as an argument that such an approach is fruitful. Thoughtful reﬂection, however, raises some additional questions. 

### 6.1 Concepts revisited, and a parable 

We have argued, contra Smith, that the approach taken by the Metathesaurus results in the construction of a set of concepts that can be regarded as universals in some signiﬁcant sense and also as reﬂecting empirical scientiﬁc reality. But we must now reconsider Smith’s criticisms from a slightly different perspective. And if the goal of introducing such concepts is to provide a representation of the meanings of terms (as is so often claimed in the Metathesaurus documentation and literature), we must wonder to precisely what degree this has been successful. Unfortunately, even if we are able to regard Metathesaurus concepts as universals, they are not very good universals. Why is this so? A metaphor or parable may be useful in illustrating the fundamental problem with Metathesaurus concepts. 

### The Parable of Weight 

Suppose that we want to develop a theory or account of the concept of weight. We know that physical objects all have some weight or other, and we know that there are a variety of relations among weights of objects. Some weights are greater than others, some weights are the same as others, weights can be measured, and weight bears certain relations to other important con­cepts (such as energy). However, in measuring the weights of objects, all that we have available to us is a set of scales that frequently disagree with one another. If object A is heavier than object B on one scale, it may be the same weight or lighter than B on another scale. We set out to “normalize” our concept of weight by constructing a scale that will result in a concept that “transcends” whatever is being measured by our variety of disagreeing scales. And we do this by constructing a new scale that measures when two objects have the same weight: when we put two objects on our scale, it says either “Same” or “Different” – which allows us to partition any set of objects into equivalence classes where the members of each class all have the same weight. This has two important consequences. 

First, it requires us to regard at least some of the results from our original scales as incorrect. This is not in itself wrong, but we must keep this result in mind when we want to make inferences that may involve mixing both the transcendent and the “local” concepts of weight that now dis­agree. 

Second, and much more important, we must understand that we have not achieved a theory or account of weight through the use of this methodology. At best, we have achieved a theory or account of the concept of same weight. We end up with a set of equivalence classes of “identically weighted objects”, but we have absolutely no information about the relations among these classes or between two objects that may be drawn from two distinct classes. We do not, for example, know that the objects in class X are all heavier than the objects in class Y, which in turn are all heavier than those in class Z. There is no “algebra of weights” or “calculus of weights” or “hierarchy of weights” that emerges from this approach, and so a signiﬁcant part of  the meaning of "weight" has not been captured.

Such is precisely the situation with Metathesaurus concepts because (look again at Deﬁnition 17) Metathesaurus concepts are constructed purely on the basis of a synonymy relation. Consequently, Metathe­saurus concepts represent a notion of same meaning, but only crudely approach representing meanings in any more signiﬁcant sense. This in turn has consequences for how successful the Metathesaurus may be in integrating multiple source vocabularies, particularly for use in knowledge discovery and inferencing. As an example, there are concepts in the Metathesaurus for Nausea (C0027497), Vomiting (C0042963), and Nausea and vomiting (C0027498), but there are no direct relations among these concepts because the only relation that the Metathesaurus recognizes between two concepts is that of synonymy, and these concepts are not synonymous with one another. There is no hierarchy of generality among concepts in the Metathesaurus, despite the fact that intuitively we recognize Nausea and vomiting as a more general concept than either Nausea or Vomiting. And there is no algebra or calculus of concepts in the Metathe­saurus. It is in this sense that while Metathesaurus concepts may be thought of as universals, they are at best rather pale and anemic universals. As a result, the concept structure of the Metathesaurus cannot represent – to any degree of precision – a concept-rich structure such as that found in SNOMED-CT. In any such attempt at representation, there will be some signiﬁcant incommensurabilities and failures to reﬂect signiﬁcant relations expressed in the richer structure.^20^

What lessons may we take from our analysis of synonymy and concepts, and from the Parable of Weight? And what are the consequences of this for the usefulness of the Metathesaurus? To begin, we must conclude that in so far as the Metathesaurus models the meaning of terms in biomedical language, it does so in only a highly restricted manner. Such claims as “In essence, [the Metathesaurus] links alterna­tive names and views of the same concept and identiﬁes useful relationships between different concepts,” (NLM (2008)) and “the UMLS Metathesaurus is a terminology integration system ... allowing for seamless mapping between terms from different terminologies through a UMLS concept” (Bodenreider (2008)) must be regarded with some degree of skepticism and understood as hyperbole. As we have seen, the Metathe­saurus does not in fact identify useful relationships between concepts (at least useful direct relationships), and the mapping between terms from different terminologies can be anything but seamless. In spite of assertions along the lines of UMLS 12, there is no structure to concepts in the Metathesaurus.^21^. The relations present in the Metathesaurus Related Concepts table suggest relationships among concepts, but in fact they are relationships among atoms and only indirectly and inexactly among concepts themselves. And while the Metathesaurus Semantic Network attempts to infuse the set of concepts with additional mean­ing, it does not (cannot) add the kind of structure to the set of concepts that is exhibited among atoms in individual sources. This is a direct result of the ambiguity of terms, the individuation of concepts by means of synonym classes, and the fact that concepts attempt to capture a transcendant sense of meaning that cannot accommodate incommensurable meanings (different senses of ‘synonymy’) across disparate sources. In the end, we must realize that Metathesaurus concepts are only vague and imprecise abstracta whose useful and precise meaning and application can be found only in the atoms that realize them, and in the relations among those atoms. Ultimately, the primary lesson to be learned here is that if synonymy is taken to be your fundamental semantic relation, much will be lost. But it hardly follows from this that the Metathesaurus is lacking in value and usefulness. 

While great care must be taken to understand precisely the relations among terms, concepts, and atoms in the Metathesaurus, and while similar care must be taken in making use of concepts as representing a transcendant sense of meaning or synonymy across multiple source vocabularies, the Metathesaurus can still contribute substantial value not only to applications of thesauri in their usage role, information retrieval role, and classiﬁcation role, but also in the knowledge discovery and inferential roles as well. Examples of the use of the Metathesaurus in the former roles are described in Bodenreider (2008), while at least a speculative scenario for the latter is sketched in Section 11 (“Applications of the referent tracking methodology”) of Smith (2006). And one non-speculative example of the use of the Metathesaurus in the knowledge discovery and data mining roles can be found in Merrill, et al. (2008a) where it serves in normalizing data ref­erences across multiple disparate large observational data bases in order to implement a new approach to drug safety analysis and evaluation. This is done by mapping terms from different medical coding schemes (e.g, ICD-9) to a representative ontology of medical conditions (currently MedDRA) and similarly mapping references to drugs (in the form of drug names and descriptions) to a representative ontology of drugs (cur­rently a modiﬁed sub-hierarchy of SNOMED-CT). The mappings are accomplished through the relations of atoms to concepts in the Metathesaurus, and by making use of some additional Metathesaurus relations. The result is a “normalized” view of multiple data bases (each of which may employ different vocabularies to represent medical conditions or drugs) to which statistical and data mining methods may then be applied in order to discover drug/condition, drug/drug, and condition/condition relations. Applications such as this demonstrate the signiﬁcant practical value that the Metathesaurus can bring to biomedical science.^22^

## Footnotes

^1^ My primary intended audience comprises current and potential users of the Metathesaurus, particularly in the areas of knowledge discovery and inferencing. Although the treatment offered here rests firmly on a technical foundation drawn from formal semantics – in the tradition of Alfred Tarski and its application in the domain of intensional logics by such contributors as Rudolph Carnap, David Kaplan, Richard Montague, Dana Scott, et al. – my intention is that it should also be readable by those who lack familiarity with such a foundation. To that end I have restricted the details of the formalism primarily to Section 4 and have taken pains to offer informal readings of the definitions and theorems together with specific examples and diagrammatic illustrations. I believe that the result will be largely intelligible to someone with knowledge of the Metathesaurus or controlled vocabularies, even if he or she is not schooled in formal semantics and model theory. I have elided proofs of theorems, but included brief hints or sketches in cases where the proof may not be wholly transparent.

^2^Even McCray and Nelson (1995), which begins with some degree of formalism and carefully defined lexical and semantic concepts, moves rapidly to a high-level view of “features” of the Metathesaurus.

^3^SNOMED-CT is the Systematized Nomenclature of Medicine - Clinical Terms, administered by the International Health Terminology Standards Development Organization.

^4^Throughout what follows, it will be necessary at times to be quite rigorous concerning the use/mention distinction: that is, to distinguish when a word or expression is being used as opposed to when it is being mentioned or referenced (vide Mates (1965), pp. 18-20). As a notational convention I will employ single quotation marks around a word or phrase to mention (refer to) that specific expression. Double quotation marks will be used in two cases: first, in the usual role of indicating direct quotations of referenced material or words used by others; and second as “scare” (sic) quotes to indicate a potentially unusual or questionable use of a term. I assume that the reader will be able to distinguish instances of use and mention within formal contexts, and thus avoid the use of such devices as quasi-quotation.

^5^Throughout what follows, it will be necessary at times to be quite rigorous concerning the use/mention distinction: that is, to distinguish when a word or expression is being used as opposed to when it is being mentioned or referenced (vide Mates (1965), pp. 18-20). As a notational convention I will employ single quotation marks around a word or phrase to mention (refer to) that specific expression. Double quotation marks will be used in two cases: first, in the usual role of indicating direct quotations of referenced material or words used by others; and second as “scare” (sic) quotes to indicate a potentially unusual or questionable use of a term. I assume that the reader will be able to distinguish instances of use and mention within formal contexts, and thus avoid the use of such devices as quasi-quotation.

^6^To be fair, I believe that at least some of the confusion surrounding the conceptual foundations of the Metathesaurus and how these are related to its knowledge representation and implementation stem from the fact that the preponderance of publications on these subjects appear in very short — typically five pages or fewer — papers that simply cannot contain the level of detail necessary to answer the questions they raise.

^7^The Metathesaurus at least implicitly attempts to provide a theory of meaning through its relations of terms to concepts and the characterization of concepts as synonym classes. But it makes no attempt to provide a theory of reference by, for example, proposing a formal model of the relation of its terms to medical entities (diseases, conditions, symptoms, drugs, procedures, etc.) in the world. This approach is, of course, wholly in keeping with the “dictionary orientation” of the Metathesaurus as a system that relates words to one another – rather than a semantic system that models the relation of words to things in the world. It is easy to sympathize with Brenner’s frustration concerning the variety of confusions and conceptual errors that often seem to accompany expositions of the Gene Ontology and similar systems. But his own note displays a rather startling naïveté and appears to propose a view of language – or at least scientific language in some sense – according to which we could not meaningfully use a term such as ‘Tetralogy of Fallot’ because it does not contain in itself a full description of the cases to which it would apply.

^8^The notion of universal has a long and sordid history in philosophy, and the word is hardly univocal. In general, however, universals (e.g., chairness) are taken to genuinely exist “in objective reality” just as particulars (e.g., this particular chair) do, and to be “instantiated” by their particulars. They are typically contrasted with concepts in that concepts are often felt to have a psychological dimension and hence not to be fully objective. However, as we shall see, it is possible to characterize an entirely non-psychologistic notion of concept that is not subject to criticisms of subjectivity. In addition, for a more sophisticated theory of concepts that renders them both non-psychologistic and distinct from universals, see Zalta (2000).

^9^But, to lend some additional confusion, note that McCray and Nelson (1995) appears to suggest a verificationist approach to meaning: “To know the meaning of a sentence is to know under what conditions (in a particular world) that sentence would be true.”

^10^The discussion here follows closely the treatments of Mates (1950) and Quine (1980b,c) which should be regarded as required reading for anyone working in the field of medical informatics, and particularly for students of the Unified Medical Language System.

^11^Such a view has also been embraced as well, in private conversation, by maintainers of the Metathesaurus.

^12^ICD-9-CM is the International Classification of Diseases, Ninth Revision, Clinical Modification, and is administered by the U.S. Center for Health Statistics and the Centers for Medical and Medicaid Services. MedDRA is the Medical Dictionary for Regulatory Activities, administered by the Maintenance and Support Services Organization.

^13^This is an area in which the structure and content of the Metathesaurus is, from the perspective of a formal language and semantics, flawed in a rather fundamental respect. A clear distinction between use and mention, and between meta- and objectlanguage, is not enforced. A symptom of this is that while most of the Metathesaurus relationship attributes apply to what can best be thought of as things in the world (diseases, drugs, laboratory tests, procedures), some of them apply instead to terms or represent semantic relations between terms and things. Examples of the latter include alias_of, expanded_form_of, has_owl_name, plain_text_form_of, tradename_of, use_for, and others. In order not to overly complicate our current approach and analysis, we will simply ignore this difficulty and assert that its consequences could be avoided through a higher degree of care and precision in representing relationship attributes and related features in the Metathesaurus.

^14^More generally, the application language must contain a set of expressions that have a certain type of semantic value. In the case of assertions or statements, this semantic value is most naturally conceived as a truth value. But in other cases it may be something else entirely. For example, if the application language is a query language, then the semantic value of a query may be something along the lines of a set of expressions (intuitively, the set of expressions “returned” by the query). In order to avoid undue complexity and generality here, we consider only application languages which have a distinctive set of expressions (often referred to as “sentences” or “formulas”) that are truth-bearers.

^15^For treatments of truth-value semantics and comparisons to referential semantics, see Leblanc (1976) and Kripke (1976).

^16^Strictly speaking, within the Metathesaurus this is complicated by the fact that what ‘term’ means in this context is not ‘distinct string’ but rather ‘lexical equivalence class of strings’ as described in UMLS 9. But for the sake of simplicity, and without loss of generality, we are ignoring this distinction throughout the discussion here.

^17^In fact, it is more complex than this and atoms are apparently individuated through a consideration of seven values associated with the term. According to Fung (2008) these are the SAB (Source Abbreviation) for the source in which the atom appears, the TTY (Term type) of that occurrence of the term, the SUI (String Unique Identifier) for the term’s string, the CODE (if any) associated with that occurrence of the term, the SDUI (Source Descriptor Unique Identifier), the SAUI (Source asserted Atom Unique Identifier), and the SCUI (Source asserted Concept Unique Identifier). Most of these attributes are not well documented (if documented at all), not all of them are available for each term, and the manner in which they are employed in creating or individuating atoms also remains undocumented. However, it is clear from this that our construal of an atom as representing a string together with a sense and a use is a reasonable approximation.

^18^There is a theological analogy here: think of the NLM as a supreme being that is omniscient concerning terms, what they denote, their uses, and their meanings. By an act of supernatural creation it then brings into existence a particular instantiation or realization of these ideas as a specific release of the Metathesaurus. But the true meanings are in the mind of the supreme being (or at least in its program code and criteria for generating atoms) – as intensions. The world is a pale reflection of the mind of the supreme being. And unlike the real supreme being, the NLM must do this on a quarterly basis.

^19^To be more precise, when we say “a concept is a rule” in this context we mean rather that a concept may be represented as or thought of as a rule for the purposes at hand – and that doing this will contribute to our understanding of concepts and their relations to other things such as terms, atoms, and synonyms. We do not mean to make any statement concerning the metaphysical nature of concepts at this point.

^20^In a Tarskian sense, the Metathesaurus as metalanguage lacks the expressive power or strength of one of its object languages.

^21^Contrast this with, for example, the very rich concept structure of Cyc which represents not only terms and concepts, but a host of semantic and other relations while not confusing linguist entities with ontological ones (Lenat and Guha (1990) and Cycorp (2008)).

^22^I thank the editor, three anonymous referees, and Ed Zalta for their careful readings of this paper and for a number of helpful suggestions that led to the clarification of several important points.
